# The Adverse Event Unit (AEU): A novel metric to measure the burden of treatment adverse events

**DOI:** 10.1371/journal.pone.0262109

**Published:** 2022-02-17

**Authors:** Michael K. Hehir, Mark Conaway, Eric M. Clark, Denise B. Aronzon, Noah Kolb, Amanda Kolb, Katherine Ruzhansky, Reza Sadjadi, Eduardo A. De Sousa, Ted M. Burns

**Affiliations:** 1 Department of Neurological Sciences, University of Vermont Larner College of Medicine, Burlington, Vermont, United States of America; 2 University of Virginia, Charlottesville, Virginia, United States of America; 3 University of Vermont Complex Systems Center, Burlington, Vermont, United States of America; 4 Timberlane Pediatrics, South Burlington, Vermont, United States of America; 5 Department of Family Medicine, University of Vermont Larner College of Medicine, Burlington, Vermont, United States of America; 6 Medical University of South Carolina, Charleston, South Carolina, United States of America; 7 Harvard Medical School Massachusetts General Hospital, Boston, Massachusetts, United States of America; 8 Mercy Clinic Neurology, Neuroscience Institute of Oklahoma City, Oklahoma City, Oklahoma, United States of America; Kaohsuing Medical University Hospital, TAIWAN

## Abstract

**Objective:**

To design a physician and patient derived tool, the Adverse Event Unit (AEU), akin to currency (e.g. U.S. Dollar), to improve AE burden measurement independent of any particular disease or medication class.

**Patients/Methods:**

A Research Electronic Data Capture (REDCap) online survey was administered to United States physicians with board certification or board eligibility in general neurology, subspecialty neurology, primary care internal medicine or family medicine, subspecialty internal medicine, general pediatrics, and subspecialty pediatrics. Physicians assigned value to 73 AE categories chosen from the Common Terminology Criteria of Adverse Events (CTCAE) relevant to neurologic disorder treatments. An online forced choice survey was administered to non-physician, potential patients, through Amazon Mechanical Turk (MTurK) to weight the severity of the same AE categories. Physician and non-physician data was combined to assign value to the AEU. Surveys completed between 1/2017 and 3/2019.

**Results:**

363 physicians rated the 73 AE categories derived from CTCAE. 660 non-physicians completed forced choice experiments comparing AEs. The AEU provides 0–10, weighted values for the AE categories studied that differ from the ordinal 1–4 CTCAE scale. For example, CTCAE severe diabetes (category 4) is assigned an AEU score of 9. Although non-physician input changed physician assigned AEU values, there was general agreement among physicians and non-physicians about severity of AEs.

**Conclusion:**

The AEU has promise to be a useful, practical tool to add precision to AE burden measurement in the clinic and in comparative efficacy research with neurology patients. AEU utility will be assessed in planned comparative efficacy clinical trials.

## Introduction

There is increasing emphasis on adverse event(AE) burden in neurology as new treatments are approved [1–3]. AEs cost more than $136 billion per year and add an average of 5 days to neurological hospitalizations [4–7]. AEs are important to patients and represent a barrier to treatment adherence. When structuring neurological treatment paradigms, among medications with equal efficacy, treatment decisions will be dictated by differences in AE burden, treatment burden, and cost. We remain without a practical metric to measure AEs that facilitates comparison of medications within and across different classes based on AEs alone.

The **Adverse Event Unit (AEU)** is a physician and patient weighted consensus unit, akin to currency (e.g. US dollar), designed to quantify and compare AE burden over time. Unlike previous measures, the AEU facilitates AE measurement independent of any disease or medication class, in terms of a number of AEUs that can be compared over time [8–13]. AEU scores can be combined with other outcome metrics and quality of life scores to better define the differences among treatments in comparative efficacy trials and in the clinic. Understanding AE tolerance in different neurological conditions and AEU validation against other disease metrics is planned for future studies. This manuscript describes the derivation of the AEU and potential applications for this new tool.

## Methods

Development of the AEU was designed as a two-phase protocol to obtain input from physician experts and potential patients. In the first phase, US physicians assigned weight to the severity of AE associated with treatments for neurological illnesses. In the second phase, non-physician potential patients recruited through the Amazon Mechanical Turk (MTurk) service (https://www.mturk.com) rated the severity of the same group of AE. Data obtained from both phases was combined to generate value for the AEU. Surveys were completed between 1/2017 and 3/2019.

### Standard protocol approvals, subject consent

The institutional review board (IRB) at the University of Vermont approved this protocol with a waiver of consent as all subjects were recruited anonymously through on-line surveys. Survey completion implied consent.

### Physician subjects

United States physicians completed an on-line survey utilizing the secure Research Electronic Data Capture tool (REDCap) [14] hosted at University of Vermont. The target population was physicians with board certification or board eligibility in general neurology, subspecialty neurology, primary care internal medicine or family medicine, subspecialty internal medicine, general pediatrics, and subspecialty pediatrics. These specialties were chosen to capture the broad range of physicians who provide medical care for neurological patients.

Champions (MKH, TMB, DBA, KR, NK, AK, and ED) identified at US centers recruited colleagues in their communities and at other centers through a combination of targeted emails and in person meetings with groups of physicians. The American Academy of Neurology facilitated recruitment of current and previous physician recipients of the development award that supported the current study. All respondents were encouraged to forward the survey to colleagues in the aforementioned medical specialties.

### Potential patient subjects

The online survey tool, MTurk, was used to recruit potential patients to represent a sample of the general population in the United States. MTurk is a viable and validated method to collect data about clinical and social science populations [15, 16]. MTurk participants produced similar results when compared to in person university recruited populations in psychological surveys, behavioral tests, matched comparison groups, economic experiments, clinical studies, and social science studies [17–20]. In general, the MTurk participants tend to be of younger age. To sample a broad age range reflective of a typical neurology patient population, we stratified the surveys into the following available age cohorts: 25–30 years, 30–35 years, 35–45 years, 45–55 years, and greater than 55 years. The MTurk tool did not permit additional age stratification in the greater than age 55 years category. Subjects were paid $5 for survey completion.

### Survey design and administration (Fig 1)

**Fig 1:**
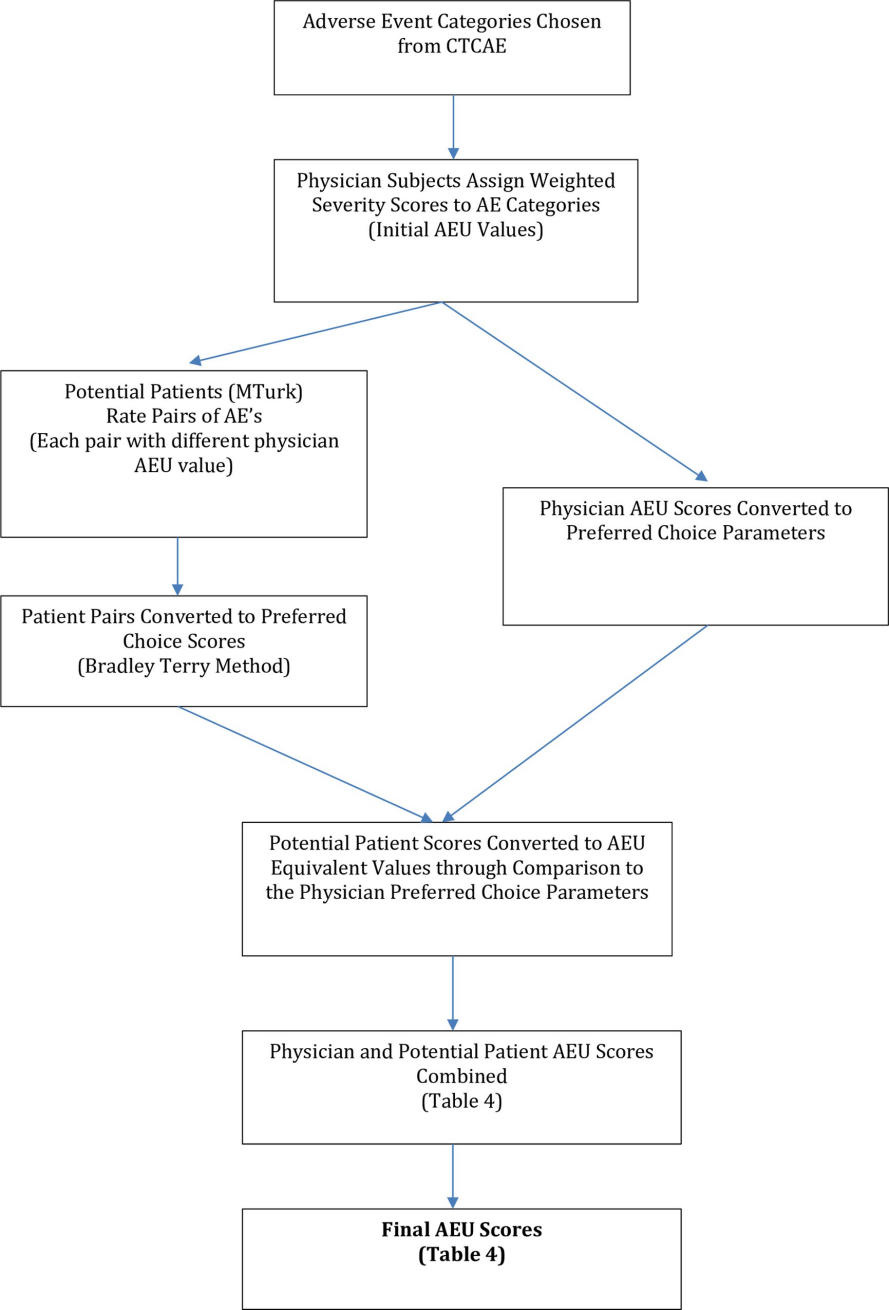
Study methodology.

#### Items for analysis

The investigators (TB and MH) chose 73 AE categories relevant to medications prescribed across the field of neurology from the Common Terminology Criteria of Adverse Events (CTCAE) version 4 for analysis (Appendix 1 in S1 File) [21]. The CTCAE is a physician expert derived, widely employed, ordinal [1–5], unweighted scale commissioned by the National Cancer Institute used to measure AE in many clinical trials [21]. Although AE severity increases along the CTCAE scale, items given the same value may not be of equal burden. For example, the AE of moderate hypertension (level 3), which carries the long-term risk of cardiovascular complications, is given the same score as a high fever of <24 hours duration (level 3). The CTCAE category 5 corresponding to death was not analyzed as we are interested in assigning value to AE that can be monitored over time while a patient undergoes treatment. The finite category of death can be measured independently without weighting because death from any cause is presumably of equal importance and consequence. Under the guidance of a board certified pediatrician (DA), items to measure congenital complications were adapted from the DSM-5 definitions of intellectual disability, an epilepsy research classification of congenital abnormalities, and neural tube defect classification systems [22–25].

#### Physician subjects

Each physician subject was asked to assign values (0 = no significance to 10 = most significant) to a random sample of 30 AEs within and across the chosen 73 CTCAE and congenital categories of varying severities. They were asked to consider each AE independent of any one disease or treatment. Subjects were also asked to factor scores they assigned both within and across AE categories as they rated AE in the survey (Appendix 2 in S1 File). A separate pediatrician survey included all the congenital malformation AE evaluated in addition to non-congenital AE. Adult physicians also rated congenital AE. Median scores with associated interquartile ranges were calculated to assign initial value to the AEU. This method of assigning weighted values to the AE categories identified from the CTCAE was adapted from method used by members of our research team (TB and MC) in the construction of the MG-Composite, a weighted, consensus, outcome measure validated for use in evaluating patients with myasthenia gravis [26].

#### Potential patient subjects

A subset of AE derived from the CTCAE and weighted by the physicians in phase 1 were converted into lay descriptions informed by Mayo Clinic descriptions of symptoms and medical conditions (https://www.mayoclinic.org/symptoms; https://www.mayoclinic.org/diseases-conditions). Potential patient friendly versions of the CTCAE have been employed in other studies [27].

Potential patient subjects reviewed pairs of AE descriptions (Table 1 and Appendix 3 in S1 File) assigned different AEU values by the physician subjects. AE pairs to review were randomly computer generated so that the compared AEs were from different AE categories and had been assigned different scores by the physician subjects. In the style of a discrete choice experiment, subjects were asked “After reviewing each pair of AE, please choose which of the two AE would be least tolerable (i.e. the most severe of the pair)” [28, 29]. They were also asked to consider: impact on quality of life (QOL), impact on life expectancy, future medical complication risk, likelihood for AE resolution following therapy change, and other factors considered important to the subject. Potential patients were not told how the physicians weighted the AE being evaluated.

**Table 1:** Example of potential patient discrete choice.

Instructions:
In this survey, you will review information about potential medication associated adverse events. You will be provided with a description of pairs of potential medication associated adverse events. Consider the side effects alone without thinking of any particular medication or disease.
**After reviewing each pair of adverse events, please choose which of the two adverse events would be least tolerable (i.e. the most severe of the pair).**
Consider the following in making your decisions:
1. Impact of the side effect on your quality of life
2. Impact on life expectancy
3. Risk to develop future medical complications because of this adverse event
4. How likely it might be the side effect will go away if medication is stopped
5. Other factors of importance to you
**Deep Vein Thrombosis (DVT):**
Deep vein thrombosis (DVT) occurs when a blood clot forms in one or more of the deep veins in your body, usually in your legs. Deep vein thrombosis can cause leg pain or swelling, but also can occur with no symptoms. Deep vein thrombosis can be very serious because blood clots in your veins can break loose and lodge in your lungs, blocking blood flow (pulmonary embolism). Treatment of DVT includes anticoagulant medications (blood thinners) and in severe circumstances, placement of a filter in your blood vessels or treatment with a clot busting medication. Blood thinner treatment increases the risk for bleeding.
Complication: (AEU 7) **
You have developed a DVT in your leg as a result of medical treatment. No complications have occurred with this DVT, such as pulmonary embolism. You require treatment with a blood thinner for at least a few months. You may have been admitted to the hospital for a short time due to this issue.
**VS**
**Headache**
Headaches may include syndromes that cause discomfort on the head including throbbing pain, stabbing pain, and numbness. Severe headaches may result in impaired physical and cognitive function. Severe headaches can impair daily function and may require treatment with medications. Drug induced headaches are likely to improve with stopping an offending medication.
Complication: (AEU 5) **
You have developed a severe headache that limits routine daily activities and self-care as a result of medical therapy. This headache lasts less than 1 week, may require a short course of pain medication (such as ibuprofen), and improves with discontinuing the offending medication. No ongoing medical therapy is required.

** Potential patient subjects were not shown the AEU value assigned by the physicians during the experiment. They are presented to illustrate that these AEs were given different weights by the physician subjects.

#### Combining physician and potential patient data

Bradley-Terry models were fit to the choices made by the potential patients using Firth’s method for penalized maximum likelihood logistic regression in SAS version 9.4 [30, 31]. The Bradley-Terry model uses the paired comparisons obtained through Mturk to estimate a set of ‘less preferred’ parameters for the AE. These parameters have the property that, if an AE with parameter A is compared to a second AE with parameter B, we would estimate that a proportion A/ (A+B) of potential patients would choose the first AE as less tolerable. Once ‘less preferred’ parameters from potential patient choices were estimated, we created integer scores by applying K-means clustering to the less preferred estimates, setting the number of clusters equal to 9 to match the range of integer scores provided by the physicians (2 to 10). Final adjustment to AEU scores was done by comparing the physician and the potential patient AEU scores. If the potential patient AEU score was greater than the physician assigned AEU, we increased the physician AEU across an entire AE category (e.g. hypertension) rating by 1. If the potential patient AEU score was less than the physician assigned AEU, we decreased the physician AEU across an entire AE category rating by 1. This method of combining physician and potential patients AEU scores was chosen to give weight to the expertise of physicians in understanding the overall short and long-term sequelae of the rated AEs. It is essential to arrive at single AEU scale to achieve the ultimate goal of developing a combined, easy to administer, best fit, weighted, consensus unit that would be feasible to administer in a clinical practice setting or clinical trial.

## Results

### Physician subjects

The targeted medical specialties were well represented (Table 2). The group was experienced and covered a wide geographic region. Recruited physicians had male and academic practice predominance. Primary care physicians practicing through university associated medical centers largely self-identified as in academic practice.

**Table 2:** Baseline characteristics.

Variable	Potential Patients	Physicians
		(n = 363)
	(n = 660)	
**Sex**, **% (n) female**	49% (325/660)	36% (129)
**Age years, % (n)**		NA
**25–30 years**	13% (79)	
**31–35 years**	10% (63)	
**36–45 years**	14% (87)	
**46–55 years**	11% (67)	
**> 55 years**	52% (314)	
**Median years practice (range)**	NA	12 (1–50)
**Academic Practice % (n)**	NA	86% (312)
**Education, % (n)**		
Grade 11 or below	1% (3)	NA
Completed Grade 12	36% (239)	NA
College/Above	63% (418)	NA
**Ethnicity, % (n)**		
Caucasian American	85% (547)	Did not ask
African American	8% (49)	
Hispanic American	4% (26)	
Asian American	3% (17)	
Native American	1% (3)	
**Geographic Region United States, % (n)**		
Northeast	22% (147)	31% (112)
Southeast	32% (210)	42% (152)
Midwest	18% (120)	12% (44)
Southwest	11% (73)	7% (26)
West	17% (109)	8% (30)
**Medical Specialty, % (n)**		
General Neurology	NA	16% (58)
Subspecialty Neurology	NA	43% (156)
Adult Primary Care	NA	17% (62)
Subspecialty Internal Medicine	NA	7% (25)
Pediatrics	NA	13% (47)
Other	NA	4% (15)

Potential patient numbers may not add to 660 because of missing values.

### Potential patient subjects

The potential patient cohort represented the wide range of ages typical of neurology patients (Table 2). The variables of geographic regions in the U.S. and sex were equally represented. Potential patients with college level of education or above were overrepresented. In addition, African Americans, Hispanic Americans, and Asian Americans were slightly underrepresented when compared to most recent U.S. Census data [32].

### Phase 1: Physician weighting

Three hundred sixty three physicians provided data from 397 surveys; 34 physicians completed two different surveys with different sets of AE. On a 0–10 scale (0 = no importance and 10 = maximal importance), physician responses ranged from 2–10 across the 73 AE categories evaluated. Median values with interquartile ranges are available in Appendix 1 in S1 File. In many circumstances, the weighted values provided by the physicians did not match the rigid 1–4 ordinal CTCAE scale. For example, the CTCAE category 1, corresponding to a mild AE for pulmonary fibrosis, received an AEU score of 6. The CTCAE category 4, corresponding to a severe AE for diabetes, received an AEU value of 9. In contrast, the severe CTCAE category 4 for headache received an AEU value of 6, similar to the AEU values assigned for CTCAE category 2 diabetes and CTCAE category 1 for pulmonary fibrosis.

### Phase 2: Potential patient forced choice

Each of 660 MTurk potential patient raters made 20 random paired AE discrete choice comparisons. Two sets of comparisons, presented to 20 participants each, could not be used because the computer randomly assigned items with same initial physician derived AEU score. These two sets of comparisons did not allow the participants to distinguish the choices, leaving 11,463 comparisons for analysis. All 73 AE categories were used in at least one paired comparison. Appendix Table 4 in S1 File provides estimates and standard errors for the logistic regression parameters estimated using Firth’s method as well as a calibration plot. The model has excellent discrimination (c-index = 0.866) and calibration. Appendix 5 in S1 File shows the results of the K-means clustering used to assign integer scores to the preference parameters. Subsequent analyses adjusted the preference parameters for demographic characteristics, age, sex, race/ethnicity, education and region of the country and of the mTurk respondents, but none of the characteristics were statistically significant, and more importantly, did not change the final ratings. Adjustment for the demographic characteristics altered the final rating in only 3 of the 73 items, and never by more than 1 point. Given the additional complexity in interpreting the results with additional covariates, we present the final ratings based on the model without adjusting for demographic characteristics.

The AE evaluated by the potential patients ranged from 2–10 AEU points on the scale generated by the physicians and the Bradley Terry method (Table 3). Severity choice values ranged from 0.33 for a mild degree of diarrhea (physician AEU 3) to 8.5 for treatment related malignancy (physician AEU 9).

**Table 3:** Combined physician and potential patient AEU values.

	Original AEU from physicians	AEU from potential patients’ choices	Final AEU rating
Diabetes	6	8	7
Osteoporosis	4	5	5
Weight Gain 6	6	4	5
Cognitive Impairment	4	4	4
Seizure	6	7	7
Heart Attack	9	9	9
Deep Vein Thrombosis (DVT)	7	7	7
Headache	5	3	4
Hallucinations 6	6	5	5
Treatment Related Malignancy (Cancer)	9	9	9
Myalgia (Muscle Pain) 6	6	2	5
Itchy Skin (Pruritus)	6	3	5
Low Platelets	5	4	4
Low Blood Sodium (Hyponatremia)	9	6	8
Flu-Like Symptoms	2	2	2
Abnormal Movements (Dyskinesia) 4	4	5	5
Kidney Stones	7	7	7
Diarrhea	4	2	3
Pulmonary Fibrosis 9	9	9	9
Hypertension (High Blood Pressure)	4	5	5
Liver Disease	3	7	4
Kidney Failure 8	8	8	8
Infection	8	6	7
Weight Loss	8	5	7
Dizziness (Vertigo)	3	3	3
Hair Loss	5	4	4
Headache 2	2	2	2
Congestive Heart Failure	5	8	6
Stroke	10	10	10
Cataracts	5	4	4
Dry Eyes	4	3	3
Diarrhea 7	7	4	6
Suicidal Thoughts	5	7	6
Dizziness (Vertigo)	5	5	5
Pancreatitis	8	8	8
Anemia (Low Red Blood Cells)	4	4	4
INR Elevation	3	4	4
Low White Blood Cells (Neutropenia)	8	6	7
Leg and Arm Swelling (Edema)	3	4	4
Constipation	6	4	5
Headache 6	6	4	5
Glaucoma 9	9	7	8
Nausea	6	3	5
Trouble Walking	5	4	4
Allergic Reaction	5	4	4
Avascular Necrosis of a Joint	8	8	8
Glaucoma 8	8	7	7
Osteoporosis	7	6	6
Stroke 6	6	7	7
Stomach Ulcer	4	5	5
Pulmonary Fibrosis 6	6	8	7
Weight Gain 3	3	3	3
Diarrhea 3	3	2	2
Hallucinations 5	5	5	5
Abnormal Movements (Dyskinesia) 6	6	7	7
Heart Rhythm Disorder (Arrhythmia)	4	5	5
Hypertension (High Blood Pressure)	5	6	6
Kidney Failure 5	5	6	6
Myalgia (Muscle Pain) 3	3	2	2
Depression	6	5	5
Fever	6	2	5
Sexual Dysfunction	6	2	5
Anxiety	5	4	4
Mania	8	6	7
Insomnia	3	3	3
Fatigue	3	2	2
Vascular Access Complications	9	8	8
Teratogen 1	10	10	10
Teratogen 2	2	6	3
Reproductive Dysfunction	4	3	3
Urine Retention	4	3	3
Heart Failure	5	9	6
Neutropenia	8	6	7

### Phase 3: Combining physician and potential patient values

Final physician and potential patient combined AEU scores are presented in Table 4. Fifty-five of the 73 items were adjusted from the originally assigned physician scores to reflect input from the potential patients (Table 3). In three categories (hallucinations, dyskinesia, and thrombosis), the physician assigned AEU value of a more severe adverse event in a category had a lower score than the immediately preceding AE. For example, moderate hallucinations had an AEU score of 6 and Severe Hallucinations/Medical Intervention Indicated (Hospitalization Not Indicated) had an AEU score of 5. This likely occurred as physicians were not shown the full range of side effects in each category when assigning scores. We did not show physicians all the AEs in a category to reduce survey burden and to prevent bias from being shown a group of side effects in a previously determined, ordinal fashion. In these three circumstances after discussion among the investigators, the decision was made to rate both categories with the higher AEU score prior to obtaining potential patient input; e.g. both hallucination categories in question have a final AEU score of 6. This decision is also supported by overlap in the interquartile range of these categories in the original physician subject weighted values.

**Table 4:** Final values Adverse Event Unit (AEU).

***Adverse Event***	***CTCAE 1***	***CTCAE 2***	***CTCAE 3***	***CTCAE 4***
***Chronic Illnesses***				
***Diabetes***	***Pre-Diabetes*:** Hemoglobin A1C: 5.7–6.4% or Fasting Glucose: 100–125mg/dL or 2H Oral Gluc Tolerance Test: 140–199 mg/dL ***4***	***Diabetes*** Hemoglobin A1C > 6.4% or Fasting Glucose > 125mg/dL or 2H Oral Gluc Tolerance Test > 200mg/dL Medical Intervention May Be Indicated ***6***	***Diabetes*** Hemoglobin A1C > 6.4% or Fasting Glucose > 125mg/dL or 2H Oral Gluc Tolerance Test > 200mg/dL AND Requiring multiple medication and/or medication escalation ***7***	***Acute Life Threatening Diabetes*** Hospitalization (eg Ketoacidosis) ***10***
***Pulmonary Fibrosis***	***Drug Related Pulmonary Fibrosis*** Mild Hypoxemia with Radiologic Pulmonary Fibrosis < 25% lung volume ***6***	***Drug Related Pulmonary Fibrosis*** Moderate Hypoxemia with Evidence of Pulmonary Hypertension OR Radiographic Evidence of Pulmonary Fibrosis 25–50% Lung Volume ***7***	***Drug Related Pulmonary Fibrosis*** Severe Hypoxemia Evidence of Right Side Heart Failure OR Radiographic Pulmonary Fibrosis > 50–75% Lung Volume ***9***	***Drug Related Pulmonary Fibrosis*** Life Threatening Consequences (e.g. hemodynamic complications) AND Intubation with Ventilatory Support ***10***
***Hypertension***	***Pre-Hypertension*** Systolic Blood Pressure: 120-139 or Diastolic Blood Pressure: 80-89 ***3***	***Hypertension*** Systolic Blood Pressure: 140-159 or Diastolic Blood Pressure: 90-99 OR Increase or Start Anti-hypertension medications ***5***	***Hypertension*** Systolic Blood Pressure: > 160 or Diastolic Blood Pressure: > 100 ***6***	***Life Threatening Hypertension*** Hospitalization for Hypertensive Urgency/Emergency ***10***
***Respiratory and Thoracic***	***Respiratory*, *Thoracic*, *Mediastinal Not Otherwise Specified*** Asymptomatic or Mild Symptoms AND Clinical or Diagnostic Observations Only ***3***	***Respiratory*, *Thoracic*, *Mediastinal Not Otherwise Specified*** Moderate, Minimal, Local, or Non-invasive Intervention Indicated AND Limiting Age Appropriate Activities Daily Living ***4***	***Respiratory*, *Thoracic*, *Mediastinal Not Otherwise Specified*** Severe or Medically Significant but Not Life Threatening AND Hospitalization or Prolong Existing Hospitalization Disabling ***8***	***Respiratory*, *Thoracic*, *Mediastinal Not Otherwise Specified*** Life Threatening Consequences AND Urgent Intervention Indicated ***9***
*Osteoporosis*	*Osteoporosis* Radiologic evidence of osteoporosis or Bone Mineral Density t score -1 to -2.5 (osteopenia) AND No intervention indicated/No loss of height *5*	***Osteoporosis*** Bone Mineral Density t score < -2.5 AND Anti-osteoporotic treatment indicated Limiting Activities Daily Living ***5***	***Osteoporosis*** Radiographic osteoporosis AND Complication not requiring hospitalization (eg fracture) ***7***	***Osteoporosis*** Radiographic osteoporosis AND Hospitalization Indicated (eg hip fracture) ***8***
***GI Ulcer***	***Gastric or Duodenal Ulcer*** Asymptomatic AND Diagnostic Observations Only Intervention Not Indicated *5*	***Gastric or Duodenal Ulcer*** Symptomatic AND Altered GI Function Medical Intervention Indicated *6*	***Gastric or Duodenal Ulcer*** Severely Altered GI Function AND TPN Indicated OR Elective Operative or Endoscopic Intervention Indicated *9*	***Gastric or Duodenal Ulcer*** Life-threatening Complications AND Urgent Operative Intervention Indicated *10*
***Endocrine***	***Endocrine Disorders*** Asymptomatic or Mild Symptoms AND Clinical or Diagnostic Observations Only *3*	***Endocrine Disorders*** Moderate Symptoms AND Minimal, Local, or Non-invasive Intervention Only *3*	***Endocrine Disorders*** Severe or Medically Significant but Not Immediately Life-threatening AND Hospitalization or Prolongation of Existing Hospitalization *8*	***Endocrine Disorders*** Life-threatening Consequences AND Urgent Intervention Indicated *9*
***Secondary Malignancy***		***Treatment Related Secondary Malignancy*** Non-Life Threatening Secondary Malignancy *7*	***Treatment Related Secondary Malignancy*** Chronic Life Threatening Secondary Malignancy AND Shortens Life Expectancy (e.g. Metastatic Disease) *9*	***Treatment Related Secondary Malignancy*** Acute Life Threatening Secondary Malignancy (e.g. blast crisis) *10*
***Renal Fail***	***Kidney Injury*** Creatinine level increase of > .3mg/dL OR Creatinine 1.5–2 times above baseline *5*	***Kidney Injury*** Creatinine 2–3 times above baseline *5*	***Kidney Injury*** Creatinine 3 times baseline or > 4mg/dL AND Hospitalization Indicated *8*	***Kidney Injury*** Life-threatening consequences AND Hospitalization, dialysis, or transplant indicated *10*
***Congestive Heart Fail***	***Heart Failure*** Asymptomatic with lab (e.g. BNP [B-natiuretic peptide]) OR Cardiac Imaging Abnormalities *6*	***Heart Failure*** Symptoms with mild to moderate activity or exertion *6*	***Heart Failure*** Severe with symptoms at rest or with minimal activity or exertion AND Intervention indicated *10*	***Heart Failure*** Life-threatening consequences AND Urgent intervention indicated(e.g. continuous IV medications, mechanical hemodynamic support *10*
***Cardiac Arrhythmia***	***Atrial or Ventricular Arrhythmia*** Asymptomatic intervention not indicated *5*	***Atrial or Ventricular Arrhythmia*** AND Non-urgent medical intervention indicated *7*	***Atrial or Ventricular Arrhythmia*** AND Acute Medical Intervention Indicated *9*	***Atrial or Ventricular Arrhythmia*** Life-threatening Consequences Hemodynamic Compromise AND Hospitalization and Urgent Intervention Indicated *10*
***Cognitive Dysfunction***	***Encephalopathy or Cognitive Dysfunction*** Mild Symptoms AND Not Interfering with Work/School/Life Performance *4*	***Encephalopathy or Cognitive Dysfunction*** Moderate Symptoms AND Interfering with Work/School/Life Performance But Capable of Independent Living *6*	***Encephalopathy or Cognitive Dysfunction*** *Severe Symptoms* AND Impairing Work/School/Life Performance *9*	***Encephalopathy or Cognitive Dysfunction*** *Life-Threatening Consequences* AND Urgent Intervention or Hospitalization Indicated *9*
***Hepatic Dysfunction***	***Functional Hepatic Impairment*** Asymptomatic or Mild Symptoms AND Clinical or Diagnostic Observation Only *4*	***Functional Hepatic Impairment*** Moderate Symptoms AND Mild, Local, or Non-Invasive Intervention *6*	***Functional Hepatic Impairment*** Severe or Medically Significant but Not Immediately Life-Threatening (e.g. Mild Encephalopathy) AND Hospitalization or Prolongation of Existing Hospitalization Indicated *9*	***Functional Hepatic Impairment*** Life-Threatening Consequences (e.g. Moderate to Severe Encephalopathy, Coma, Hemorrhage) AND Hepatic Transplant and Urgent Hospitalization Indicated *10*
***Seizures***	***Drug Related Seizure*** Brief Partial Seizure No Loss of Consciousness *6*	***Drug Related Seizure*** Brief Generalized Seizure with Loss of Consciousness *7*	***Drug Related Seizure*** Multiple Seizures Despite Medical Intervention *10*	***Drug Related Seizure*** Life-Threatening Prolonged Repetitive Seizures (Status Epilepticus) AND Requiring Hospitalization and Urgent Intervention *10*
***Glaucoma***	***Glaucoma*** Elevated Intraocular Pressure (EIOP) without Visual Field Deficits AND Single Topical Agent Indicated *3*	***Glaucoma*** Elevated Intraocular Pressure (EIOP) with Early Visual Field Deficit AND Multiple Topical Agents and/or Oral Agent Indicated *5*	***Glaucoma*** Elevated Intraocular Pressure (EIOP) with Marked Visual Field Deficit AND Operative Intervention Indicated *7*	***Glaucoma*** Blindness in Affected Eye (20/200 or Worse) *8*
***Cataracts***	***Cataracts*** Asymptomatic AND Clinical or Diagnostic Observation Only *2*	***Cataracts*** Symptomatic AND Moderate Decrease in Visual Acuity (20/40 or better) *4*	***Cataracts*** Symptomatic with Marked Decrease Visual Acuity (worse than 20/40 but better than 20/200) AND Operative Intervention Indicated (e.g. cataract surgery) *6*	***Cataracts*** Blindness (20/200 or worse) in affected eye *7*
***Acute Coronary Syndrome***	***Acute Coronary Syndrome*** Symptomatic Progressive Angina AND Cardiac Enzymes Normal Hemodynamically Stable *7*	***Acute Coronary Syndrome*** Symptomatic Unstable Angina OR Acute Myocardial Infarction AND Cardiac Enzymes Abnormal Hemodynamically Stable *8*	***Acute Coronary Syndrome*** Symptomatic Unstable Angina OR Acute Myocardial Infarction AND Cardiac Enzymes Abnormal Hemodynamically Unstable *9*	***Acute Coronary Syndrome*** Life Threatening Consequences Hemodynamically Unstable AND ICU Level Care Indicated *10*
***Stroke***	***Stroke*** Asymptomatic or Mild Neurologic Deficit Radiographic Findings Only *7*	***Stroke*** Moderate Neurologic Deficit *9*	***Stroke*** Severe Neurologic Deficit Prolonged Hospitalization AND/OR Requires Care in Long-term Facility *10*	***Stroke*** Life-Threatening Consequences If Survive Requires Prolonged Use of Tracheostomy AND/OR Percutaneous Gastrostomy Tube Requires Care in Long-Term Facility *10*
***Anxiety***	***Anxiety*** *Mild symptoms; intervention not indicated* *1*	***Anxiety*** *Moderate symptoms; limiting instrumental ADL* *4*	***Anxiety*** *Severe symptoms; limiting self-care ADL; hospitalization not indicated* *6*	***Anxiety*** *Life-threatening; hospitalization indicated* *8*
***Depression***	***Depression*** *Mild depressive symptoms* *2*	***Depression*** *Moderate depressive symptoms; limiting instrumental ADL* *5*	***Depression*** *Severe depressive symptoms; limiting self-care ADL; hospitalization not indicated* *6*	***Depression*** *Life-threatening consequences*, *threats of harm to self or others; hospitalization indicated* *8*
***Mania***	***Mania*** *Mild manic symptoms (e*.*g*., *elevated mood*, *rapid thoughts*, *rapid speech*, *decreased need for sleep)* *4*	***Mania*** *Moderate manic symptoms (e*.*g*., *relationship and work difficulties; poor hygiene)* *6*	***Mania*** *Severe manic symptoms (e*.*g*., *hypomania; major sexual or financial indiscretions); hospitalization not indicated* *9*	***Mania*** *Life-threatening consequences*, *threats of harm to self or others; hospitalization indicated* *9*
***Adverse Events*** ***Typically Shorter Duration***				
***Fever***	***Drug Fever*** Temperature 38–39C (100.4–102.2F) < 24Hours ***2***	***Drug Fever*** Temperature 39–40C (102.2–104F) < 24 Hours ***2***	***Drug Fever*** Temperature > 40C for < 24Hours ***5***	***Drug Fever*** Temperature > 40C for > 24Hours ***5***
***Headache***	***Headache*** Mild Pain < 1 week *2*	***Headache*** Moderate Pain AND Limits Activities Daily Living< 1 week *2*	***Headache*** Severe Pain AND Limits Self Care and Activities Daily Living < 1 week *4*	***Chronic Headache*** Moderate to Severe Pain Duration > 1 week *5*
***Infection***	***Infection*** Asymptomatic or Mild Symptoms Diagnostic intervention only Medical intervention not indicated *1*	***Infection*** Symptomatic AND Minimal, local, or non-invasive intervention indicated *2*	***Infection*** Severe of medically significant but not immediately life threatening AND Hospitalization or prolongation existing hospitalization *7*	***Infection*** Life threatening consequences AND Urgent hospitalization or ICU level care indicated *9*
***Weight Gain***	***Weight Gain*** 5-10% increase from baseline *3*	***Weight Gain*** 10-20% increase from baseline *4*	***Weight Gain*** > 20% increase from baseline *6*	
***Weight Loss***	***Weight Loss*** 5-10% Decrease from Baseline *1*	***Weight Loss*** 10-20% Decrease from Baseline AND Requires Nutritional Support *4*	***Weight Loss*** > 20% Decrease from Baseline AND Tube feed or TPN Indicated *7*	
***Thrombosis***	***Venous Thrombosis*** (e.g. superficial thrombosis) *3*	***Venous Thrombosis*** Uncomplicated deep vein thrombosis AND Medical Intervention Indicated *7*	***Uncomplicated Pulmonary Embolism or*** Non-embolic Cardiac Mural Thrombus AND Medical Intervention Indicated. *7*	***Life Threatening Thrombotic Event*** (e.g. Complicated Pulmonary Embolism, Arterial Insufficiency) Hemodynamic Instability AND Urgent Intervention Indicated *10*
***Avascular Necrosis***	***Avascular Necrosis*** Asymptomatic Clinical or Diagnostic Interventions Only *4*	***Avascular Necrosis*** Symptomatic Limiting Instrumental Activities Daily Living *6*	***Avascular Necrosis*** Severe Symptoms Limiting Self Care Activities Daily Living AND Operative Intervention Indicated *8*	***Avascular Necrosis*** Life Threatening Consequences AND Urgent Intervention Indicated *9*
***Hair Loss***	***Alopecia*** Hair Loss < 50% of Normal for Individual Only Noticeable from Close Inspection Different Hair Style but Doesn’t Require Wig to Camouflage *3*	***Alopecia*** Hair Loss >/= 50% of Normal for Individual May be associated with Psychosocial Impact *4*		
***Diarrhea***	***Diarrhea*** Increase < 4 Stools Per Day Over Baseline OR Mild Increase in Ostomy Output Compared to Baseline *2*	***Diarrhea*** Increase 4–6 Stools Per Day Over Baseline Moderate Increase in Ostomy Output Compared to Baseline AND Limiting Instrumental Activities Daily Living *3*	***Diarrhea*** Increase < 7 Stools Per Day Over Baseline Incontinence OR Severe Increase in Ostomy Output Compared to Baseline AND Hospitalization Indicated *6*	***Diarrhea*** Life-threatening Complications AND Hospitalization Indicated *8*
***Nausea***	***Nausea*** Loss of Appetite Without Alteration in Eating Habits *1*	***Nausea*** Oral Intake Decreased AND Without Weight Loss, Dehydration, or Malnutrition *2*	***Nausea*** Vomiting or Anti-Emetics Required *5*	***Nausea*** Inadequate Oral Caloric or Fluid Intake OR Refractory Vomiting with Tube Feed, TPN *7*
***Vertigo***	***Vertigo*** Mild symptoms *3*	***Vertigo*** Moderate Symptoms AND Limiting Instrumental Activities Daily Living *5*	***Vertigo*** Severe Symptoms AND Limiting Self Care *7*	
***Vascular Access***	***Vascular Access*** Device Dislodgement, Blockage, Leak, Malposition AND Device Replacement Indicated *3*	***Vascular Access*** Deep Vein or Cardiac Thrombosis AND Intervention Indicated (e.g. anticoagulation, lysis, filter, invasive procedure) *6*	***Vascular Access*** Embolic Event Related to Vascular Access(e.g. pulmonary embolism or life threatening thrombus) *8*	
***Suicidal Ideation***	***Suicidal Ideation*** Increased Thoughts of Death But No Wish to Kill Oneself *7*	***Suicidal Ideation*** Suicidal Ideation with No Specific Plan or Intent *7*	***Suicidal Ideation*** Specific Plan to Commit Suicide OR Suicide Attempt without Serious Intent to Die May Not Require Hospitalization *10*	***Suicidal Ideation*** Suicide Attempt with Intent to Die OR Specific Plan to Commit Suicide with Serious Intent to Die Requires Hospitalization *10*
***Hallucinations***	***Hallucinations*** Mild Hallucinations (e.g. perceptual distortions) *5*	***Hallucinations*** Moderate Hallucinations *7*	***Hallucinations*** Severe Hallucinations AND Medical Intervention Indicated Hospitalization Not Indicated *7*	***Hallucinations*** Life-threatening Complications Threats of Harm to Self or Others AND Hospitalization Indicated *9*
***SICCA***	***Dry Mouth*** Symptomatic (e.g. dry thick saliva) AND Without Significant Dietary Alteration *1*	***Dry Mouth*** Moderate Symptoms AND Oral Intake Alterations (e.g. copious water, other lubricants) OR Diet Limited to Purees *3*	***Dry Mouth*** Inadequate Oral Intake AND Tube Feeds, TPN Indicated *7*	
***Cushingoid***	***Cushingoid*** Mild Symptoms AND Intervention Not Indicated *3*	***Cushingoid*** Moderate Symptoms AND Medical Intervention Indicated *6*	***Cushingoid*** Severe Symptoms AND Medical Intervention or Hospitalization Indicated *7*	
***Myalgia***	***Drug Related Myalgia*** Mild Pain *1*	***Drug Related Myalgia*** Moderate Pain AND Limiting Instrumental Activities Daily Living *5*	***Drug Related Myalgia*** Severe Pain AND Limiting Self Care Activities Daily Living *5*	
***Pruritus***	***Pruritus (Itching)*** Mild or Localized AND Topical Intervention Indicated *1*	***Pruritus (Itching)*** Intense or Widespread Intermittent OR Skin Changes from Scratching AND Oral Intervention Indicated *3*	***Pruritus (Itching)*** Intense or Widespread, Constant AND Oral Corticosteroid or Immunosuppressive Therapy Indicated *5*	
***Dermatologic***	***Skin Disorders*** Asymptomatic or Mild Symptoms Clinical or Diagnostic Observations Only *1*	***Skin Disorders*** Moderate, Minimal, Local, or Non-Invasive Intervention Indicated *2*	***Skin Disorders*** Severe or Medically Significant but Not Immediately Life Threatening AND Hospitalization or Prolongation of Existing Hospitalization *6*	***Skin Disorders*** Life-threatening Consequences AND Urgent Intervention Indicated *7*
***Dry Eye***	***Dry Eye*** Mild symptoms relieved by lubricants *1*	***Dry Eye*** Multiple agents indicated to relieve symptoms AND Limiting instrumental activities of daily living *3*	***Dry Eye*** Decrease in visual acuity (worse than 20/40) AND Limiting self-care activities of daily living *5*	
***Constipation***	***Drug Related Constipation*** New mild symptoms Occasional use of stool softeners, laxatives, dietary modification, or enema. *1*	***Drug Related Constipation*** Persistent symptoms with regular use of laxatives or enemas AND Limiting instrumental activities of daily living *3*	***Drug Related Constipation*** *Obstipation with manual evacuation indicated* AND Limiting self-care activities of daily living *5*	***Drug Related Constipation*** *Life threatening consequences AND* Hospitalization indicated *8*
***Infusion Site Reaction***	***Injection Site Reaction*** Tenderness with or without associated symptoms(e.g. warmth, erythema, itching) *2*	***Injection Site Reaction*** Pain, Lipodystrophy, Edema, Phlebitis *4*	***Injection Site Reaction*** Ulceration or Necrosis with Severe Tissue Damage AND Operative Intervention Indicated *8*	***Injection Site Reaction*** Life-threatening consequences AND Urgent intervention indicated *9*
***Falls***	***Drug Related Fall*** Minor with no resultant injuries Intervention not indicated *2*	***Drug Related Fall*** Symptomatic AND Non-invasive Intervention Indicated *4*	***Drug Related Fall*** Hospitalization Indicated *6*	
***Allergic Reaction***	***Allergic Reaction*** Transient Flushing AND No Intervention Indicated *1*	***Allergic Reaction*** Intervention or Infusion Indicated AND Responds Quickly to Medications Prophylaxis < 24 Hours *4*	***Allergic Reaction*** Prolonged (Not rapidly responsive to medical intervention) AND Recurrence of Symptoms Following Medical Treatment Hospitalization Indicated *5*	***Allergic Reaction*** Life Threatening Consequences AND Urgent ICU Level Care Indicated (e.g. Stevens Johnson Syndrome, Anaphylaxis, Angioedema) *9*
***Sexual Dysfunction***	***Sexual Dysfunction*** Mild Sexual Dysfunction Not Adversely Affecting Relationship *2*	***Sexual Dysfunction*** Moderate Sexual Dysfunction AND Adversely Affecting Relationship *5*	***Sexual Dysfunction*** Severe Increase in Sexual Interest Leading to Dangerous Behavior *7*	
***Edema***	***Edema Limbs*** Asymptomatic or Mild Symptoms Clinical or Diagnostic Observations Only *3*	***Edema Limbs*** Moderate Symptoms AND Minimal, Local, or Non-Invasive Intervention Indicated *4*	***Edema Limbs*** Severe or Medically Significant but Not Life-Threatening AND Hospitalization or Prolongation Existing Hospitalization Indicated *8*	
***Fatigue***	***Fatigue*** Fatigue Relieved By Rest *2*	***Fatigue*** Fatigue Not Relieved by Rest *2*	***Fatigue*** Fatigue Not Relieved by Rest AND Limiting Self Care Activities Daily Living *5*	
***Anemia***	***Anemia*** Hemoglobin (Hbg) < Lower Limit Normal - 10 g/dL OR Hgb < Lower Limits Normal - 6.2mmol/L OR Hgb < Lower Limits Normal - 100g/L *3*	***Anemia*** Hemoglobin (Hbg) < 10- 8 g/dL OR Hgb < 6.2–4.9 mmol/L OR Hgb < 100–80g/L *4*	***Anemia*** Hemoglobin (Hbg) < 8 g/dL OR Hgb < 4.9 mmol/L OR Hgb < 80 g/L AND Transfusion Indicated *8*	***Anemia*** Life-Threatening Consequences AND Urgent Intervention Indicated *9*
***DIC***	***Disseminated Intravascular Coagulation*** Lab Findings with No Bleeding *5*	***Disseminated Intravascular Coagulation*** Lab Findings with Bleeding *9*	***Disseminated Intravascular Coagulation*** Life-Threatening Consequences AND Hospitalization and Urgent Intervention Indicated *10*	
***Dyskinesia***	***Dyskinesia*** Mild Restlessness or Increased Motor Activity *5*	***Dyskinesia*** Moderate Restlessness or Increased Motor Activity AND Limiting Instrumental Activities Daily Living *8*	***Dyskinesia*** Severe Restlessness or Increased Motor Activity AND Limiting Self Care Activities Daily Living *8*	
***Kidney Stones***	***Renal Calculi (Kidney Stones)*** Asymptomatic of Mild Symptoms AND Occasional Use of Non-Prescription Agents *3*	***Renal Calculi (Kidney Stones)*** Symptomatic AND Oral Anti-emetics OR Around the Clock Non-Prescription Analgesics or Any Oral Narcotic *6*	***Renal Calculi (Kidney Stones)*** Hospitalization Indicated AND IV Intervention Elective Endoscopic or Radiographic Intervention Indicated *7*	***Renal Calculi (Kidney Stones)*** Life-Threatening Complications AND Urgent Endoscopic or Operative Intervention and Hospitalization Indicated *9*
***Insomnia***	***Insomnia*** Mild Difficulty Falling Asleep, Staying Asleep, or Waking Up Early *3*	***Insomnia*** Moderate Difficulty Falling Asleep, Staying Asleep, or Waking Up Early *3*	***Insomnia*** Severe Difficulty Falling Asleep, Staying Asleep, or Waking Up Early *6*	
***Pancreatitis***	***Pancreatitis*** Enzyme Elevation or Radiologic Findings Only *3*	***Pancreatitis*** Severe Pain, Vomiting AND Medical Intervention Indicated (e.g. analgesia, nutritional support) *8*	***Pancreatitis*** Life-Threatening Consequences AND Hospitalization and Urgent Intervention Indicated *9*	
***Flu Reaction***	***Flu Like Symptoms*** Mild Flu-Like Symptoms *2*	***Flu Like Symptoms*** Moderate Flu-Like Symptoms > 1 day *2*	***Flu Like Symptoms*** Severe Flu-Like Symptoms > 1 Day AND Limiting Self Care Activities Daily Living *5*	
***Gait Dysfunction***	***Gait Disturbance*** Mild Change in Gait (e.g. wide based, limping, or hobbling) *4*	***Gait Disturbance*** Moderate Change in Gait (e.g. wide based, limping, or hobbling) AND Assistive Device Indicated *4*	***Gait Disturbance*** Severe Change in Gait AND Disabling Requires Wheelchair *8*	
***Febrile Neutropenia***	***Febrile Neutropenia*** ANC < 1000/mm3 with Single Temp > 38.3C (101F) OR Sustained Temp >/= 38C (100.4) for more than 1 Hour *6*	***Febrile Neutropenia*** Life-Threatening Consequences AND Hospitalization and Urgent Intervention Indicated *8*		
***Laboratory Abnormalities***				
***INR Elevation***	***INR Increase*** INR > 1–1.5 x Upper Limit Normal OR INR > 1–1.5 x Above Baseline if on Anticoagulation *4*	***INR Increase*** INR > 1.5–2.5 x Upper Limit Normal OR INR > 1.5–2.5 x Above Baseline if on Anticoagulation *6*	***INR Increase*** INR > 2.5 x Upper Limit Normal OR INR > 2.5 x Above Baseline if on Anticoagulation *8*	
***ALT/AST Elevation***	***ALT or AST Elevation*** Lab 2–3 x Upper Limit Normal *3*	***ALT or AST Elevation*** Lab 3–5 x Upper Limit Normal *4*	***ALT or AST Elevation*** Lab 5 -20 x Upper Limit Normal *7*	***ALT or AST Elevation*** Lab > 20 x Upper Limit Normal *8*
***Neutropenia***	***Neutrophil Count Reduced*** ANC < Lower Limit Normal - 1500/mm3 OR ANC < Lower Limit Normal - 1.5 x 10e9/L *3*	***Neutrophil Count Reduced*** ANC < 1500–1000/mm3 OR ANC < 1.5–1 x 10e9/L *4*	***Neutrophil Count Reduced*** ANC < 1000–500/mm3 OR ANC < 1–0.5 x 10e9/L *7*	***Neutrophil Count Reduced*** ANC < 500/mm3 OR ANC < 0.5 x 10e9/L *7*
***Low Platelets***	***Platelet Count Reduced*** Platelets < Lower Limit Normal - 75,000/mm3 OR Platelets < Lower Limit Normal - 75 x 10e9/L *4*	***Platelet Count Reduced*** Platelets < 75,000–50,000/mm3 OR Platelets < 75–50 x 10e9/L *6*	***Platelet Count Reduced*** Platelets < 50,000–25,000/mm3 OR Platelets < 50–25 x 10e9/L *6*	***Platelet Count Reduced*** Platelets < 25,000/mm3 OR Platelets < 25 x 10e9/L *7*
***Hypernatremia***	***Hypernatremia*** Na > Upper Limit Normal - 150 mmol/L *3*	***Hypernatremia*** Na > 150–155 mmol/L *3*	***Hypernatremia*** Na > 155–160 mmol/L AND Hospitalization Indicated *7*	***Hypernatremia*** Na > 160 mmol/L and Life Threatening Consequences Hospitalization Indicated *7*
***Hyponatremia***	***Hyponatremia*** Na < 130 mmol/L - Lower Limit Normal *2*	***Hyponatremia*** Na < 120–130 mmol/L *4*	***Hyponatremia*** Na < 120 mmol/L Life Threatening Complications *8*	
***Hypokalemia***	***Hypokalemia*** K < 3 mmol/L - Lower Limit Normal *3*	***Hypokalemia*** K < 3 mmol/L - Lower Limit Normal AND Symptomatic Intervention Indicated *5*	***Hypokalemia*** K < 2.5–3 mmol/L AND Hospitalization Indicated *5*	***Hypokalemia*** K < 2.5 mmol/L with Life Threatening Consequences AND Urgent Hospitalization *8*
***Hyperkalemia***	***Hyperkalemia*** K > Upper Limits Normal - 5.5 mmol/L *2*	***Hyperkalemia*** K > 5.5–6 mmol/L *5*	***Hyperkalemia*** K > 6–7 mmol/L *5*	***Hyperkalemia*** K > 7 mmol/L *8*

***Reproductive and*** ***Congenital Complications***							
***Reproductive System Disorder*** ***(Adult Male/Female)***	***Reproductive System Disorders*** Asymptomatic or mild symptoms Clinical or Diagnostic Observations Only Intervention not indicated *2*	***Reproductive System Disorders*** Moderate AND Minimal, Local, or Non-Invasive Intervention *3*	***Reproductive System Disorders*** Severe AND Hospitalization or Prolongation of Existing Hospitalization *6*	***Reproductive System Disorders*** Sterilization of Patient at Child Bearing Age(Male or Female) *8*	***Reproductive System Disorders*** Life-threatening Consequences AND Urgent Intervention Indicated *9*		
***Congenital Abnormalities***	***Minor Physical Congenital Abnormalities with No Clinical Significance*** (e.g. single palmar crease, pre-auricular skin tag) *3*	***Intrauterine Growth Restriction*:** Birth weight below 10^*th*^ *percentile for gestational age*. *Requires additional monitoring*. *May have clinical consequences*. *8*	***Physical Features Persisting in a Term Infant that are Typically Only Present Before 37 Weeks Gestation*** (e.g. patent ductus arteriosis, undescended testicle) *6*	***Genetic Disorders*:** Genetic disorders compatible with survival but likely to result in disability. (e.g. Chromosomal Disorders, Trisomy 21) *10*	***Facial/Orthopaedic Sequelae*:** Hypoplasia of face or fingers, Arthrogryposis, Loss of Limb *10*	***Microcephaly/Macrocephaly*:** Abnormalities in head circumference and brain size. Requires additional monitoring. May result in cognitive dysfunction. *10*	***Major Cardiac*, *Pulmonary*, *Gastrointestinal Malformations*:** Structural abnormality with surgical or medical *importance*. (e.g. major cyanotic heart disease, esophageal atresia, gastroschisis) *10*
***Spina Bifida***	***Closed Spinal Dysraphism (i*.*e*. *spina bifida occulta)*:** Failure of fusion of vertebral bodies. Unexposed neural tissue. Skin Intact. 8	***Myelomenigocele*:** Cleft in vertebral column *9*	***Encephalocele*:** Protrusion of brain and/or meninges through skull. Covered by skin. AND May be compatible with survival but with likely disability. *10*	***Anencephaly*:** Open deficit, cranial tube exposed. Not compatible with survival. *10*			
***Teratogenicity***	***Teratogenic Mild Intellectual Disability*:** Children require academic supports to learn skills *appropriate for age*. *Social skills and personal judgment immature for age*. *Most individuals independent in daily living activities*, *employable in jobs*, *and able to live independently*. *9*	***Teratogenic Moderate Intellectual Disability*:** Conceptual and academic skills lag well behind peers. Adults able attain elementary level skills. Social cues, judgment, and life decisions require support. Most capable of personal care with support (e.g. group home). *10*	***Teratogenic Severe Intellectual Disability*:** Little understanding of written language, numbers, time, money concepts. Caretakers provide extensive support. Benefit from interaction with family/familiar people with limited social interaction. Trainable in some basic activities of daily living. *10*	***Teratogenic Profound Intellectual Disability*:** May use objects in a goal directed fashion for self-care or recreation. May understand some gestures and emotional cues. Dependent on support for all activities of daily living. *10*			

## Discussion

In the age of precision medicine, well-designed, practical outcome measures and decision support tools expand the data we track about patients to better inform medical decisions [33, 34]. Unlike previous measures, the physician and potential patient derived AEU quantifies AE burden in a common currency independent of any disease or medication class, that can be compared among different medications over time. The AEU may facilitate movement from more gestalt AE burden measurement to more precise AE burden measurement, enriching treatment discussions between patients and physicians.

Individual patients and physicians may not value AE in the same way, e.g. patients with more severe conditions such as cancer, may tolerate a higher burden of AE. As a consensus metric, the AEU is not designed to be an absolute measure of burden and distress for any particular patient but rather a way to keep the AE burden score. The AEU is designed to best estimate the market price of specific AEs, similarly to how the price is set for a good or service, e.g. $10 for a basketball and $30,000 for a car. Consumers decide if they are willing to pay consensus prices for these goods. Similarly, patients can be given AEU scores corresponding to the number and type of AEs they develop on a given therapy. In combination with measures of disease improvement, financial burden, overall QOL, severity of a patient’s medical condition, patient age, and other factors unique to a particular patient, patients can decide whether they are willing to tolerate a specific AEU burden when making treatment decisions with their physician. Future validation projects, like one underway in a population of patients with myasthenia gravis, will attempt to understand clinically meaningful differences in AEU score over time for different patient populations.

Attempts have been made to develop disease and medication specific measures of AE [8–13]. Disease and medication specificity limit broad applicability. Quality Adjusted Life Year (QALY) is a useful measure of population cost effectiveness of varied treatments [35, 36]. Since the QALY encompasses all aspects of health, financial cost, and QOL, it cannot measure AE burden alone. As a population based tool, the QALY is a less practical way to measure treatment burden in a comparative efficacy trial or in the clinic.

The CTCAE is a medication independent, physician derived AE measurement tool [21]. Due to lack of weighting and patient input, it provides only granular AE burden measurement. We built the AEU based on the strengths of the CTCAE. The diverse physician group incorporated a wide range of opinions about AE impact on overall health accounting for both current effects (e.g. joint pain) as well as future secondary consequences (e.g. stroke due to new diabetes) to assign AEU values. Although all physicians surveyed could rate congenital complication AEs, all pediatricians surveyed weighted these items as they care for impacted children.

The AEU incorporates potential patient opinions in assigning AE burden values. The use of potential patients rather than patients with particular diagnoses allows AE burden to be scored independent of any particular disease or medication. While we were not able to stratify the sample by whether MTurk respondents were parents, many subjects who rated congenital AEs self-identified as parents in the comment section. Since MTurk doesn’t permit stratification by ethnicity, some groups were slightly underrepresented in our sample. We observed even representation of U.S. geographic regions. Utilizing MTurk, we obtained hundreds of opinions within days of survey release. Although MTurk introduced bias due to requirement of basic computing skills, it reduced other bias, including the selection bias of clinicians when choosing patients for participation in research. We found recruitment through this online tool to be a logistical and cost-effective strategy to easily obtain opinions from large samples. This method has the potential to be a powerful method for studies like this one and to obtain preliminary data for clinical study design while reducing the inherent bias of the small focus group method.

We believe the weighted consensus AEU values provides a more complete measurement of AE burden. A CTCAE category 1 is often classified as mild [37]. However, all CTCAE categories across different AEs are not of equal value and were not weighted the same among our cohort. A CTCAE grade 1 may not reflect a good outcome in all circumstances. For example, CTCAE Grade 1 pulmonary fibrosis, received an AEU score of 7 and was rated the same as CTCAE Grade 4 osteoporosis (Table 4). We also believe the AEU’s independence of any particular disease or medication class is essential to allow comparison of treatments across medication classes. For example, prednisone and IVIG, treatments with different AE profiles, could be compared by the AEU in patients with myasthenia gravis.

Although 75% of AE categories required final AEU value adjustment when physician and potential patient values were combined, only 12% of items had a rating difference of 3 or more points between physicians and potential patients (Table 4). This suggests that while there is difference in physician and potential patient opinions on AE severity, there appears to be general agreement among the groups. Use of the Bradley-Terry paired comparisons model was a useful way to put the physician ratings, collected as scores, and the MTurk ratings, collected as a sequence of paired comparisons, on the same scale. Although physician opinions anchored AEU values, potential patient opinions were incorporated via the discrete choice surveys. We believe adjusting the AEU score to incorporate opinions of both groups strengthens the future applicability of this tool. In practice, patients often rely on physician expert caregivers to guide medical decisions.

We believe the AEU has great promise to be a useful, practical tool to add precision to AE burden measurement in the clinic and in comparative efficacy research for neurology patients. Future studies may show the AEU to be useful in other medical specialties. In comparative efficacy research, we anticipate that AE burden of drugs from different classes can be compared by AEU burden. Assigning an AEU score over time will account for more transient AEs that drop out over time (e.g. single headache) and more persistent AEs (e.g. new hypertension). The AEU score can be combined with other disease specific outcome metrics and QOL metrics to measure differences among medications over time. Evaluation of the validity, utility, and value of the AEU in comparative efficacy trials in myasthenia gravis and other neurological disorders is under way. If the AEU is useful in these studies, translation of some or all of the other items in the CTCAE could be performed to generalize the AEU to other medical subspecialties.

## Supporting information

S1 File (DOCX)

S2 File Physician subject raw data form 2. (CSV)

S3 File All potential patient choices raw data. (XLSX)

S4 File Physician subject raw data form 5. (CSV)

S5 File AEU item codes raw data. (XLSX)

S6 File Physician subject raw data form 1. (CSV)

S7 File Physician subject raw data pediatric form. (CSV)

S8 File Physician subject raw data form 6. (CSV)

S9 File Physician subject raw data form 4. (CSV)

S10 File Physician subject raw data form 3. (CSV)

## Abbreviations

AE: Adverse Events
AEU: Adverse Event Unit
CTCAE: Common Terminology of Adverse Events
IRB: Institutional Review Board
MTurk: Amazon Mechanical Turk
QALY: Quality Adjusted Life Year
QOL: Quality of Life
REDCap: Research Electronic Data Capture
US: United States

## References

[1] Smith AG. The Cost of Rare Diseases is Threatening the U.S. Health Care System. Harvard Business Review. 2017.

[2] Schepelmann K, Winter Y, Spottke AE, Claus D, Grothe C, Schroder R, et al. Socioeconomic burden of amyotrophic lateral sclerosis, myasthenia gravis and facioscapulohumeral muscular dystrophy. Journal of neurology. 2010;257(1):15–23. doi: 10.1007/s00415-009-5256-6 19629566

[3] Guptill JT, Marano A, Krueger A, Sanders DB. Cost analysis of myasthenia gravis from a large U.S. insurance database. Muscle & nerve. 2011;44(6):907–11. doi: 10.1002/mus.22212 22102461

[4] Classen DC, Pestotnik SL, Evans RS, Lloyd JF, Burke JP. Adverse drug events in hospitalized patients. Excess length of stay, extra costs, and attributable mortality. JAMA: the journal of the American Medical Association. 1997;277(4):301–6. 9002492

[5] Bates DW, Spell N, Cullen DJ, Burdick E, Laird N, Petersen LA, et al. The costs of adverse drug events in hospitalized patients. Adverse Drug Events Prevention Study Group. JAMA: the journal of the American Medical Association. 1997;277(4):307–11. 9002493

[6] Quality AfHRa. Reducing and Preventing Adverse Drug Events to Decrease Hospital Costs. https://archiveahrqgov/research/findings/factsheets/errors-safety/aderia/adehtml. 2001.

[7] Martinez-Ramirez D, Giugni JC, Little CS, Chapman JP, Ahmed B, Monari E, et al. Missing dosages and neuroleptic usage may prolong length of stay in hospitalized Parkinson’s disease patients. PloS one. 2015;10(4):e0124356. doi: 10.1371/journal.pone.0124356 25884484 PMC4401689

[8] Testa MA, Anderson RB, Nackley JF, Hollenberg NK. Quality of life and antihypertensive therapy in men. A comparison of captopril with enalapril. The Quality-of-Life Hypertension Study Group. The New England journal of medicine. 1993;328(13):907–13. doi: 10.1056/NEJM199304013281302 8446137

[9] Katz NP, Mou J, Trudeau J, Xiang J, Vorsanger G, Orman C, et al. Development and preliminary validation of an integrated efficacy-tolerability composite measure for the evaluation of analgesics. Pain. 2015;156(7):1357–65. doi: 10.1097/j.pain.0000000000000186 25867124

[10] Mohr DC, Likosky W, Boudewyn AC, Marietta P, Dwyer P, Van der Wende J, et al. Side effect profile and adherence to in the treatment of multiple sclerosis with interferon beta-1a. Multiple sclerosis. 1998;4(6):487–9. doi: 10.1177/135245859800400605 9987757

[11] Voruganti L, Cortese L, Oyewumi L, Cernovsky Z, Zirul S, Awad A. Comparative evaluation of conventional and novel antipsychotic drugs with reference to their subjective tolerability, side-effect profile and impact on quality of life. Schizophrenia research. 2000;43(2–3):135–45. doi: 10.1016/s0920-9964(99)00154-1 10858632

[12] Smith SM, Wang AT, Katz NP, McDermott MP, Burke LB, Coplan P, et al. Adverse event assessment, analysis, and reporting in recent published analgesic clinical trials: ACTTION systematic review and recommendations. Pain. 2013;154(7):997–1008. doi: 10.1016/j.pain.2013.03.003 23602344

[13] Hunsinger M, Smith SM, Rothstein D, McKeown A, Parkhurst M, Hertz S, et al. Adverse event reporting in nonpharmacologic, noninterventional pain clinical trials: ACTTION systematic review. Pain. 2014;155(11):2253–62. doi: 10.1016/j.pain.2014.08.004 25123543

[14] Harris PA, Taylor R, Thielke R, Payne J, Gonzalez N, Conde JG. Research electronic data capture (REDCap)—a metadata-driven methodology and workflow process for providing translational research informatics support. Journal of biomedical informatics. 2009;42(2):377–81. doi: 10.1016/j.jbi.2008.08.010 18929686 PMC2700030

[15] Behrend TS, Sharek DJ, Meade AW, Wiebe EN. The viability of crowdsourcing for survey research. Behavior research methods. 2011;43(3):800–13. doi: 10.3758/s13428-011-0081-0 21437749

[16] Goodman JK CC, Cheema A. Data collection in a flat world: the strengths and weaknesses of mechanical turk samples. Journal of Behavioral Decision Making. 2013;26:213–24.

[17] Schmidt GB, Jettinghoff WM. Using Amazon Mechanical Turk and other compensated crowdsourcing sites. Bus Horizons. 2016;59(4):391–400.

[18] Paolacci G, Chandler J. Inside the Turk: Understanding Mechanical Turk as a Participant Pool. Current Directions in Psychological Science. 2014;23(3):184–8.

[19] Buhrmester M, Kwang T, Gosling SD. Amazon’s Mechanical Turk: A New Source of Inexpensive, Yet High-Quality, Data? Perspectives on psychological science: a journal of the Association for Psychological Science. 2011;6(1):3–5. doi: 10.1177/1745691610393980 26162106

[20] Bernstein J, Calamia M. Characteristics of a Mild Traumatic Brain Injury Sample Recruited Using Amazon’s Mechanical Turk. PM & R: the journal of injury, function, and rehabilitation. 2017. doi: 10.1016/j.pmrj.2017.06.010 28633999

[21] Institute NC. Common Terminology Criteria for Adverse Events v4.0. NIH Publication 09–7473. 2009.

[22] Association AP. Neurodevelopmental Disorders. Diagnostic and statistical manual of mental disorders (5th ed)2013.

[23] Holmes LB, Harvey EA, Coull BA, Huntington KB, Khoshbin S, Hayes AM, et al. The teratogenicity of anticonvulsant drugs. The New England journal of medicine. 2001;344(15):1132–8. doi: 10.1056/NEJM200104123441504 11297704

[24] Copp AJ, Stanier P, Greene ND. Neural tube defects: recent advances, unsolved questions, and controversies. The Lancet Neurology. 2013;12(8):799–810. doi: 10.1016/S1474-4422(13)70110-8 23790957 PMC4023229

[25] Greene ND, Copp AJ. Neural tube defects. Annual review of neuroscience. 2014;37:221–42. doi: 10.1146/annurev-neuro-062012-170354 25032496 PMC4486472

[26] Burns TM, Conaway MR, Cutter GR, Sanders DB, Muscle Study G. Construction of an efficient evaluative instrument for myasthenia gravis: the MG composite. Muscle & nerve. 2008;38(6):1553–62. doi: 10.1002/mus.21185 19016543

[27] Basch E, Iasonos A, McDonough T, Barz A, Culkin A, Kris MG, et al. Patient versus clinician symptom reporting using the National Cancer Institute Common Terminology Criteria for Adverse Events: results of a questionnaire-based study. The Lancet Oncology. 2006;7(11):903–9. doi: 10.1016/S1470-2045(06)70910-X 17081915

[28] Reed Johnson F, Lancsar E, Marshall D, Kilambi V, Muhlbacher A, Regier DA, et al. Constructing experimental designs for discrete-choice experiments: report of the ISPOR Conjoint Analysis Experimental Design Good Research Practices Task Force. Value in health: the journal of the International Society for Pharmacoeconomics and Outcomes Research. 2013;16(1):3–13. 23337210 10.1016/j.jval.2012.08.2223

[29] Louviere JL, Flynn T.N., and Carson R.T. Discrete Choice Experiments and Not Conjoint Analysis. Journal of Choice Modelling. 2010;3(3):57–72.

[30] Firth D. Bias reduction of maximum likelihood estimates. Biometrika. 1993;80(1):27–38.

[31] Hunter D. MM Algorithms for Generalized Bradley-Terry Models. Annals of Statistics. 2004;32(1):384–406.

[32] Bureau USC. Race and Hispanic Origin 2010 [Available from: https://www.census.gov/quickfacts/fact/table/US/PST045217#qf-headnote-a].

[33] Mirnezami R, Nicholson J, Darzi A. Preparing for precision medicine. The New England journal of medicine. 2012;366(6):489–91. doi: 10.1056/NEJMp1114866 22256780

[34] Burns TM. The best of both worlds: Using patient-reported plus physician-scored measures during the evaluation of myasthenia gravis. Muscle & nerve. 2016;53(1):3–4. doi: 10.1002/mus.24953 26506220

[35] Bravo Vergel Y, Sculpher M. Quality-adjusted life years. Practical neurology. 2008;8(3):175–82. doi: 10.1136/pn.2007.140186 18502950

[36] Sassi F. Calculating QALYs, comparing QALY and DALY calculations. Health policy and planning. 2006;21(5):402–8. doi: 10.1093/heapol/czl018 16877455

[37] Sanders DB, Wolfe GI, Benatar M, Evoli A, Gilhus NE, Illa I, et al. International consensus guidance for management of myasthenia gravis: Executive summary. Neurology. 2016;87(4):419–25. doi: 10.1212/WNL.0000000000002790 27358333 PMC4977114

