# Supplementary material for: The Adverse Event Unit (AEU): A novel metric to measure the burden of treatment adverse events
Source: PLoS One. 2022 Feb 17;17(2):e0262109. doi: 10.1371/journal.pone.0262109 (PMC8853570; doi:10.1371/journal.pone.0262109)

**Appendix 1:**

**Physician Derived AEU Values**

| **Adverse Event**  Median (Interquartile Range) | **CTCAE 1** | **CTCAE 2** | **CTCAE 3** | **CTCAE 4** |  |
| --- | --- | --- | --- | --- | --- |
| **Chronic Illnesses** |  |  |  |  |  |
| **Diabetes** | **Pre-Diabetes:**  Hemoglobin A1C: 5.7 - 6.4% or Fasting Glucose: 100 - 125mg/dL or  2H Oral Gluc Tolerance Test: 140 - 199 mg/dL  **3 (2-5)** | **Diabetes**  Hemoglobin A1C > 6.4% or Fasting Glucose > 125mg/dL or  2H Oral Gluc Tolerance Test > 200mg/dL  Medical Intervention May Be Indicated  **5 (4-7)** | **Diabetes**  Hemoglobin A1C > 6.4% or Fasting Glucose > 125mg/dL or  2H Oral Gluc Tolerance Test > 200mg/dL AND Requiring multiple medication and/or medication escalation  **6 (5-8)** | **Acute Life Threatening Diabetes**  Hospitalization (eg Ketoacidosis)  **9 (8-10)** |  |
| **Pulmonary Fibrosis** | **Drug Related Pulmonary Fibrosis**  Mild Hypoxemia with Radiologic Pulmonary Fibrosis < 25% lung volume  **6 (5-7)** | **Drug Related Pulmonary Fibrosis**  Moderate Hypoxemia with Evidence of Pulmonary Hypertension OR  Radiographic Evidence of Pulmonary Fibrosis 25 - 50% Lung Volume  **7 (6-8)** | **Drug Related Pulmonary Fibrosis**  Severe Hypoxemia  Evidence of Right Side Heart Failure OR   Radiographic Pulmonary Fibrosis > 50 - 75% Lung Volume  **9 (9-10)** | **Drug Related Pulmonary Fibrosis**  Life Threatening Consequences (e.g. hemodynamic complications)  AND  Intubation with Ventilatory Support  **10 (9-10)** |  |
| **Hypertension** | **Pre-Hypertension** Systolic Blood Pressure: 120-139 or Diastolic Blood Pressure: 80-89  **2 (1-2)** | **Hypertension** Systolic Blood Pressure: 140-159 or Diastolic Blood Pressure: 90-99 OR Increase or Start Anti-hypertension medications  **4 (3-5)** | **Hypertension** Systolic Blood Pressure: > 160 or Diastolic Blood Pressure: > 100  **5 (4-6)** | **Life Threatening Hypertension** Hospitalization for Hypertensive Urgency/Emergency  **9 (8-10)** |  |
| **Respiratory and Thoracic** | **Respiratory, Thoracic, Mediastinal Not Otherwise Specified**  Asymptomatic or Mild Symptoms AND Clinical or Diagnostic Observations Only  3 (2-4) | **Respiratory, Thoracic, Mediastinal Not Otherwise Specified**  Moderate, Minimal, Local, or Non-invasive Intervention Indicated AND Limiting Age Appropriate Activities Daily Living  4 (3-6) | **Respiratory, Thoracic, Mediastinal Not Otherwise Specified**  Severe or Medically Significant but Not Life Threatening AND Hospitalization or Prolong Existing Hospitalization Disabling  8 (7-9) | **Respiratory, Thoracic, Mediastinal Not Otherwise Specified**  Life Threatening Consequences AND Urgent Intervention Indicated  9 (8-10) |  |
| **Osteoporosis** | **Osteoporosis**  Radiologic evidence of osteoporosis or  Bone Mineral Density t score -1 to -2.5 (osteopenia)  AND  No intervention indicated/No loss of height  4 (3-5) | **Osteoporosis**  Bone Mineral Density t score < -2.5   AND  Anti-osteoporotic treatment indicated  Limiting Activities Daily Living  4 (3-5) | **Osteoporosis**  Radiographic osteoporosis   AND  Complication not requiring hospitalization (eg fracture)  7 (6-8) | **Osteoporosis**  Radiographic osteoporosis   AND  Hospitalization Indicated (eg hip fracture)  8 (7-8.5) |  |
| **GI Ulcer** | **Gastric or Duodenal Ulcer**  Asymptomatic AND Diagnostic Observations Only  Intervention Not Indicated  4 (3-5) | **Gastric or Duodenal Ulcer**  Symptomatic  AND Altered GI Function  Medical Intervention Indicated  5 (3-6) | **Gastric or Duodenal Ulcer**  Severely Altered GI Function AND TPN Indicated OR  Elective Operative or Endoscopic Intervention Indicated  8 (8-9) | **Gastric or Duodenal Ulcer**  Life-threatening Complications AND Urgent Operative Intervention Indicated  9 (8-10) |  |
| **Endocrine** | **Endocrine Disorders**  Asymptomatic or Mild Symptoms AND  Clinical or Diagnostic Observations Only  3 (2-4) | **Endocrine Disorders**  Moderate Symptoms AND  Minimal, Local, or Non-invasive Intervention Only  3 (2-4) | **Endocrine Disorders**  Severe or Medically Significant but Not Immediately Life-threatening  AND  Hospitalization or Prolongation of Existing Hospitalization  8 (7-8) | **Endocrine Disorders**  Life-threatening Consequences AND  Urgent Intervention Indicated  8.5 (7-9) |  |
| **Secondary Malignancy** | **Treatment Related Secondary Malignancy**  Non-Life Threatening Secondary Malignancy  7 (5-8) | **Treatment Related Secondary Malignancy**  Chronic Life Threatening Secondary Malignancy AND Shortens Life Expectancy (e.g. Metastatic Disease)  9 (9-10) | **Treatment Related Secondary Malignancy**  Acute Life Threatening Secondary Malignancy (e.g. blast crisis)  10 (9-10) |  |  |
| **Renal Fail** | **Kidney Injury**  Creatinine level increase of > .3mg/dL OR  Creatinine 1.5 - 2 times above baseline  5 (4-6) | **Kidney Injury**  Creatinine 2 - 3 times above baseline  5 (4.5-6) | **Kidney Injury**  Creatinine 3 times baseline or > 4mg/dL AND  Hospitalization Indicated  8 (7-10) | **Kidney Injury**  Life-threatening consequences AND   Hospitalization, dialysis, or transplant indicated  10 (9-10) |  |
| **Congestive Heart Fail** | **Heart Failure**  Asymptomatic with lab (e.g. BNP [B-natiuretic peptide]) OR  Cardiac Imaging Abnormalities  5 (4-6) | **Heart Failure**  Symptoms with mild to moderate activity or exertion  5 (4-7) | **Heart Failure**  Severe with symptoms at rest or with minimal activity or exertion AND  Intervention indicated  9 (8-10) | **Heart Failure**  Life-threatening consequences AND  Urgent intervention indicated(e.g. continuous IV medications, mechanical hemodynamic support  10 (9-10) |  |
| **Cardiac Arrhythmia** | **Atrial or Ventricular Arrhythmia**   Asymptomatic intervention not indicated  4 (3-6) | **Atrial or Ventricular Arrhythmia**   AND  Non-urgent medical intervention indicated  6 (5-8) | **Atrial or Ventricular Arrhythmia**  AND   Acute Medical Intervention Indicated  8 (6-8) | **Atrial or Ventricular Arrhythmia**   Life-threatening Consequences Hemodynamic Compromise  AND  Hospitalization and Urgent Intervention Indicated  10 (9-10) |  |
| **Reproductive System Disorder**  **(Adult Male/Female)** | **Reproductive System Disorders**  Asymptomatic or mild symptoms  Clinical or Diagnostic Observations Only  Intervention not indicated  3 (2-3.5) | **Reproductive System Disorders**  Moderate AND  Minimal, Local, or Non-Invasive Intervention  4 (3-5) | **Reproductive System Disorders**  Severe AND   Hospitalization or Prolongation of Existing Hospitalization  7 (6-8) | **Reproductive System Disorders**  Sterilization of Patient at Child Bearing Age(Male or Female)  9 (8-10) | **Reproductive System Disorders**  Life-threatening Consequences AND  Urgent Intervention Indicated  9.5 (8-10) |
| **Cognitive Dysfunction** | **Encephalopathy or Cognitive Dysfunction**  Mild Symptoms AND Not Interfering with Work/School/Life Performance  4 (3-5) | **Encephalopathy or Cognitive Dysfunction**  Moderate Symptoms AND Interfering with Work/School/Life Performance But Capable of Independent Living  6 (5-7) | **Encephalopathy or Cognitive Dysfunction**  Severe Symptoms AND Impairing Work/School/Life Performance  9 (8-9) | **Encephalopathy or Cognitive Dysfunction**  Life-Threatening Consequences AND Urgent Intervention or Hospitalization Indicated  9 (8-10) |  |
| **Hepatic Dysfunction** | **Functional Hepatic Impairment**  Asymptomatic or Mild Symptoms AND Clinical or Diagnostic Observation Only  3 (2-4) | **Functional Hepatic Impairment**  Moderate Symptoms AND Mild, Local, or Non-Invasive Intervention  5 (3-6) | **Functional Hepatic Impairment**  Severe or Medically Significant but Not Immediately Life-Threatening (e.g. Mild Encephalopathy)  AND  Hospitalization or Prolongation of Existing Hospitalization Indicated  8 (7-9) | **Functional Hepatic Impairment**  Life-Threatening Consequences (e.g. Moderate to Severe Encephalopathy, Coma, Hemorrhage)  AND  Hepatic Transplant and Urgent Hospitalization Indicated  10 (9-10) |  |
| **Seizures** | **Drug Related Seizure**  Brief Partial Seizure  No Loss of Consciousness  5 (4-7) | **Drug Related Seizure**  Brief Generalized Seizure with  Loss of Consciousness  6 (5-8) | **Drug Related Seizure**  Multiple Seizures Despite Medical Intervention  9 (9-10) | **Drug Related Seizure**  Life-Threatening Prolonged Repetitive Seizures (Status Epilepticus) AND Requiring Hospitalization and Urgent Intervention  10 (9-10) |  |
| **Glaucoma** | **Glaucoma**  Elevated Intraocular Pressure (EIOP) without Visual Field Deficits AND Single Topical Agent Indicated  4 (3-6) | **Glaucoma**  Elevated Intraocular Pressure (EIOP) with Early Visual Field Deficit AND Multiple Topical Agents and/or Oral Agent Indicated  6 (5-7) | **Glaucoma**  Elevated Intraocular Pressure (EIOP) with Marked Visual Field Deficit AND Operative Intervention Indicated  8 (7-10) | **Glaucoma**  Blindness in Affected Eye (20/200 or Worse)  9 (9-10) |  |
| **Cataracts** | **Cataracts**  Asymptomatic AND  Clinical or Diagnostic Observation Only  3 (2-5) | **Cataracts**  Symptomatic AND  Moderate Decrease in Visual Acuity (20/40 or better)  5 (4-5) | **Cataracts**  Symptomatic with Marked Decrease Visual Acuity (worse than 20/40 but better than 20/200) AND Operative Intervention Indicated (e.g. cataract surgery)  7 (6-8) | **Cataracts**  Blindness (20/200 or worse) in affected eye  8 (7-9) |  |
| **Acute Coronary Syndrome** | **Acute Coronary Syndrome**  Symptomatic Progressive Angina AND Cardiac Enzymes Normal Hemodynamically Stable  7 (5-8) | **Acute Coronary Syndrome**  Symptomatic Unstable Angina OR Acute Myocardial Infarction AND Cardiac Enzymes Abnormal Hemodynamically Stable  8 (6-8) | **Acute Coronary Syndrome**  Symptomatic Unstable Angina OR Acute Myocardial Infarction AND Cardiac Enzymes Abnormal Hemodynamically Unstable  9 (9-10) | **Acute Coronary Syndrome**  Life Threatening Consequences Hemodynamically Unstable AND ICU Level Care Indicated  10 (9-10) |  |
| **Stroke** | **Stroke**  Asymptomatic or Mild Neurologic Deficit  Radiographic Findings Only  6 (5-8) | **Stroke**  Moderate Neurologic Deficit  8 (7-9) | **Stroke**  Severe Neurologic Deficit  Prolonged Hospitalization AND/OR   Requires Care in Long-term Facility  10 (9-10) | **Stroke**  Life-Threatening Consequences  If Survive Requires Prolonged Use of Tracheostomy AND/OR Percutaneous Gastrostomy Tube Requires Care in Long-Term Facility  10 (9-10) |  |
| **Anxiety** | **Anxiety**  Mild symptoms; intervention not indicated  2 (1-4) | **Anxiety**  Moderate symptoms; limiting instrumental ADL  5 (4.5-5.5) | **Anxiety**  Severe symptoms; limiting self care ADL; hospitalization not indicated  7 (6-8) | **Anxiety**  Life-threatening; hospitalization indicated  9 (9-10) |  |
| **Depression** | **Depression**  Mild depressive symptoms  3 (2-3) | **Depression**  Moderate depressive symptoms; limiting instrumental ADL  6 (4-6.5) | **Depression**  Severe depressive symptoms; limiting self care ADL; hospitalization not indicated  7 (7-8) | **Depression**  Life-threatening consequences, threats of harm to self or others; hospitalization indicated  9 (9-10) |  |
| **Mania** | **Mania**  Mild manic symptoms (e.g., elevated mood, rapid thoughts, rapid speech, decreased need for sleep)  5 (4.5-6) | **Mania**  Moderate manic symptoms (e.g., relationship and work difficulties; poor hygiene)  7 (5-7) | **Mania**  Severe manic symptoms (e.g., hypomania; major sexual or financial indiscretions); hospitalization not indicated  8 (8-10) | **Mania**  Life-threatening consequences, threats of harm to self or others; hospitalization indicated  9 (9-10) |  |

| **Congenital Complications** |  |  |  |  |  |  |  |  |
| --- | --- | --- | --- | --- | --- | --- | --- | --- |
| **Congenital Abnormalities** | **Minor Physical Congenital Abnormalities with No Clinical Significance**  (e.g. single palmar crease, pre-auricular skin tag)  2 (1-4) | **Intrauterine Growth Restriction:**  Birth weight below 10th percentile for gestational age. Requires additional monitoring. May have clinical consequences.  7 (5-9) | **Physical Features Persisting in a Term Infant that are Typically Only Present Before 37 Weeks Gestation**  (e.g. patent ductus arteriosis, undescended testicle)  5 (4-7) | **Genetic Disorders:**  Genetic disorders compatible with survival but likely to result in disability.  (e.g. Chromosomal Disorders, Trisomy 21)  10 (8-10) | **Facial/Orthopaedic Sequelae:**  Hypoplasia of face or fingers, Arthrogryposis, Loss of Limb  9 (7-10) | **Microcephaly/Macrocephaly:**  Abnormalities in head circumference and brain size. Requires additional monitoring. May result in cognitive dysfunction.  9 (8-10) | **Major Cardiac, Pulmonary, Gastrointestinal Malformations:**  Structural abnormality with surgical or medical importance.  (e.g. major cyanotic heart disease, esophageal atresia, gastroschisis)  10 (9-10) |  |
| **Spina Bifida** | **Closed Spinal Dysraphism (i.e. spina bifida occulta):**  Failure of fusion of vertebral bodies. Unexposed neural tissue. Skin Intact.  7 (4-9) | **Myelomenigocele:**  Cleft in vertebral column  8 (7-9) | **Encephalocele:**  Protrusion of brain and/or meninges through skull. Covered by skin.   AND  May be compatible with survival but with likely disability.  10 (9-10) | **Anencephaly:**  Open deficit, cranial tube exposed. Not compatible with survival.  10 (10) |  |  |  |  |
| **Teratogenicity** | **Teratogenic Mild Intellectual Disability:**  Children require academic supports to learn skills appropriate for age. Social skills and personal judgment immature for age. Most individuals independent in daily living activities, employable in jobs, and able to live independently.  7.5 (6.5-8.5) | **Teratogenic Moderate Intellectual Disability:**  Conceptual and academic skills lag well behind peers. Adults able attain elementary level skills. Social cues, judgment, and life decisions require support. Most capable of personal care with support (e.g. group home).  9 (8-10) | **Teratogenic Severe Intellectual Disability:**  Little understanding of written language, numbers, time, money concepts. Caretakers provide extensive support. Benefit from interaction with family/familiar people with limited social interaction. Trainable in some basic activities of daily living.  10 (10-10) | **Taratogenic Profound Intellectual Disability:**  May use objects in a goal directed fashion for self-care or recreation. May understand some gestures and emotional cues. Dependent on support for all activities of daily living.  10 (9-10) |  |  |  |  |
|  |  |  |  |  |  |  |  |  |

| **Adverse Events**  **Typically Shorter Duration** |  |  |  |  |
| --- | --- | --- | --- | --- |
| **Fever** | **Drug Fever**  Temperature 38 - 39C (100.4 - 102.2F) < 24Hours  **3 (2-4)** | **Drug Fever**  Temperature 39 - 40C (102.2 - 104F) < 24 Hours  **3 (2-5)** | **Drug Fever**  Temperature > 40C for < 24Hours  **6 (4-8)** | **Drug Fever**  Temperature > 40C for > 24Hours  **6 (4-8)** |
| **Headache** | **Headache**  Mild Pain < 1 week  2 (1-3) | **Headache**  Moderate Pain AND Limits Activities Daily Living< 1 week  3 (2-5) | **Headache**  Severe Pain AND Limits Self Care and Activities Daily Living < 1 week  5 (4-6) | **Chronic Headache**  Moderate to Severe Pain Duration > 1 week  6 (5-7) |
| **Infection** | **Infection**  Asymptomatic or Mild Symptoms  Diagnostic intervention only Medical intervention not indicated  2 (1-3) | **Infection**  Symptomatic AND  Minimal, local, or non-invasive intervention indicated  3 (2.5-4) | **Infection**  Severe of medically significant but not immediately life threatening AND Hospitalization or prolongation existing hospitalization  8 (7-9) | **Infection**  Life threatening consequences AND Urgent hospitalization or ICU level care indicated  10 (9-10) |
| **Weight Gain** | **Weight Gain**  5-10% increase from baseline  3 (2-3) | **Weight Gain**  10-20% increase from baseline  4 (3-6) | **Weight Gain**  > 20% increase from baseline  6 (5-7) |  |
| **Weight Loss** | **Weight Loss**  5-10% Decrease from Baseline  2 (1-3) | **Weight Loss**  10-20% Decrease from Baseline AND Requires Nutritional Support  5 (3-6.5) | **Weight Loss**  > 20% Decrease from Baseline AND Tube feed or TPN Indicated  8 (7-9) |  |
| **Thrombosis** | **Venous Thrombosis**  (e.g. superficial thrombosis)  3 (2-4) | **Venous Thrombosis**  Uncomplicated deep vein thrombosis  AND  Medical Intervention Indicated  7 (6-8) | **Uncomplicated Pulmonary Embolism or  Non-embolic Cardiac Mural Thrombus**  AND  Medical Intervention Indicated.  6 (5-7) | **Life Threatening Thrombotic Event**  (e.g. Complicated Pulmonary Embolism, Arterial Insufficiency)  Hemodynamic Instability AND Urgent Intervention Indicated  10 (9-10) |
| **Avascular Necrosis** | **Avascular Necrosis**  Asymptomatic Clinical or Diagnostic Interventions Only  4.5 (3.5-6) | **Avascular Necrosis**  Symptomatic Limiting Instrumental Activities Daily Living  6 (5-7) | **Avascular Necrosis**  Severe Symptoms  Limiting Self Care Activities Daily Living AND Operative Intervention Indicated  8.5 (7.5-9.5) | **Avascular Necrosis**  Life Threatening Consequences AND Urgent Intervention Indicated  9 (8-10) |
| **Hair Loss** | **Alopecia**  Hair Loss < 50% of Normal for Individual Only Noticeable from Close Inspection Different Hair Style but Doesn't Require Wig to Camouflage  3.5 (2-5) | **Alopecia**  Hair Loss >/= 50% of Normal for Individual  May be associated with Psychosocial Impact  5 (4-6) |  |  |
| **Diarrhea** | **Diarrhea**  Increase < 4 Stools Per Day Over Baseline OR  Mild Increase in Ostomy Output Compared to Baseline  3 (3-4) | **Diarrhea**  Increase 4 - 6 Stools Per Day Over Baseline  Moderate Increase in Ostomy Output Compared to Baseline AND Limiting Instrumental Activities Daily Living  4 (3.5-5) | **Diarrhea**  Increase < 7 Stools Per Day Over Baseline  Incontinence OR Severe Increase in Ostomy Output Compared to Baseline AND Hospitalization Indicated  7 (6-9) | **Diarrhea**  Life-threatening Complications AND  Hospitalization Indicated  9 (8-10) |
| **Nausea** | **Nausea**  Loss of Appetite Without Alteration in Eating Habits  2 (2-4) | **Nausea**  Oral Intake Decreased  AND Without Weight Loss, Dehydration, or Malnutrition  3 (2-3) | **Nausea**  Vomiting or Anti-Emetics Required  6 (5-7) | **Nausea**  Inadequate Oral Caloric or Fluid Intake OR Refractory Vomiting with Tube Feed, TPN  8 (7-9) |
| **Vertigo** | **Vertigo**  Mild symptoms  3 (2-3) | **Vertigo**  Moderate Symptoms AND Limiting Instrumental Activities Daily Living  5 (4-6) | **Vertigo**  Severe Symptoms AND Limiting Self Care  7 (6-8) |  |
| **Vascular Access** | **Vascular Access**  Device Dislodgement, Blockage, Leak, Malposition  AND Device Replacement Indicated  4.5 (3-6) | **Vascular Access**  Deep Vein or Cardiac Thrombosis  AND Intervention Indicated (e.g. anticoagulation, lysis, filter, invasive procedure)  7 (6-8) | **Vascular Access**  Embolic Event Related to Vascular Access(e.g. pulmonary embolism or life threatening thrombus)  9 (8-10) |  |
| **Suicidal Ideation** | **Suicidal Ideation**  Increased Thoughts of Death But No Wish to Kill Oneself  6 (5-7) | **Suicidal Ideation**  Suicidal Ideation with No Specific Plan or Intent  5 (4-7) | **Suicidal Ideation**  Specific Plan to Commit Suicide OR Suicide Attempt without Serious Intent to Die May Not Require Hospitalization  9 (9-10) | **Suicidal Ideation**  Suicide Attempt with Intent to Die OR Specific Plan to Commit Suicide with Serious Intent to Die  Requires Hospitalization  9 (8-10) |
| **Hallucinations** | **Hallucinations**  Mild Hallucinations (e.g. perceptual distortions)  5 (4-7) | **Hallucinations**  Moderate Hallucinations  7 (6-8) | **Hallucinations**  Severe Hallucinations AND Medical Intervention Indicated Hospitalization Not Indicated  6 (5-7) | **Hallucinations**  Life-threatening Complications Threats of Harm to Self or Others AND Hospitalization Indicated  9 (8-10) |
| **SICCA** | **Dry Mouth** Symptomatic (e.g. dry thick saliva) AND Without Significant Dietary Alteration  2 (1-3) | **Dry Mouth** Moderate Symptoms AND  Oral Intake Alterations (e.g. copious water, other lubricants) OR Diet Limited to Purees  4 (2-6) | **Dry Mouth**  Inadequate Oral Intake AND Tube Feeds, TPN Indicated  8 (6-9) |  |
| **Cushingoid** | **Cushingoid**  Mild Symptoms AND  Intervention Not Indicated  3 (2-5) | **Cushingoid**  Moderate Symptoms AND  Medical Intervention Indicated  6 (5-7) | **Cushingoid**  Severe Symptoms AND  Medical Intervention or Hospitalization Indicated  7 (6-8) |  |
| **Myalgia** | **Drug Related Myalgia**  Mild Pain  2 (2-3) | **Drug Related Myalgia**  Moderate Pain AND  Limiting Instrumental Activities Daily Living  6 (4-7) | **Drug Related Myalgia**  Severe Pain AND  Limiting Self Care Activities Daily Living  6 (5-8) |  |
| **Pruritus** | **Pruritus (Itching)**  Mild or Localized AND  Topical Intervention Indicated  2 (2-3) | **Pruritus (Itching)**  Intense or Widespread Intermittent OR Skin Changes from Scratching AND Oral Intervention Indicated  4 (2-4) | **Pruritus (Itching)**  Intense or Widespread, Constant AND Oral Corticosteroid or Immunosuppressive Therapy Indicated  6 (4-7) |  |
| **Dermatologic** | **Skin Disorders**  Asymptomatic or Mild Symptoms  Clinical or Diagnostic Observations Only  2 (1-3) | **Skin Disorders**  Moderate, Minimal, Local, or Non-Invasive Intervention Indicated  3 (2-4) | **Skin Disorders**  Severe or Medically Significant but Not Immediately Life Threatening  AND Hospitalization or Prolongation of Existing Hospitalization  7 (6-8) | **Skin Disorders**  Life-threatening Consequences AND  Urgent Intervention Indicated  8.5 (8-9) |
| **Dry Eye** | **Dry Eye**  Mild symptoms relieved by lubricants  2 (1-3) | **Dry Eye**  Multiple agents indicated to relieve symptoms AND Limiting instrumental activities of daily living  4 (2-5) | **Dry Eye**  Decrease in visual acuity (worse than 20/40) AND Limiting self care activities of daily living  6 (5.5-8) |  |
| **Constipation** | **Drug Related Constipation**  New mild symptoms Occasional use of stool softeners, laxatives, dietary modification, or enema.  2 (1-3) | **Drug Related Constipation**  Persistent symptoms with regular use of laxatives or enemas AND Limiting instrumental activities of daily living  4 (3-5) | **Drug Related Constipation**  Obstipation with manual evacuation indicated AND Limiting self-care activities of daily living  6 (5-7) | **Drug Related Constipation**  Life threatening consequences AND Hospitalization indicated  9 (7.5-10) |
| **Infusion Site Reaction** | **Injection Site Reaction**  Tenderness with or without associated symptoms(e.g. warmth, erythema, itching)  2 (1-3) | **Injection Site Reaction**  Pain, Lipodystrophy, Edema, Phlebitis  4 (2.5-5) | **Injection Site Reaction**  Ulceration or Necrosis with Severe Tissue Damage AND Operative Intervention Indicated  8 (7-9) | **Injection Site Reaction**  Life-threatening consequences AND Urgent intervention indicated  9 (8-10) |
| **Falls** | **Drug Related Fall**  Minor with no resultant injuries Intervention not indicated  3 (2.5-4) | **Drug Related Fall**  Symptomatic AND Non-invasive Intervention Indicated  5 (4-5) | **Drug Related Fall**  Hospitalization Indicated  7 (6.5-8) |  |
| **Allergic Reaction** | **Allergic Reaction**  Transient Flushing AND No Intervention Indicated  2 (1-3) | **Allergic Reaction**  Intervention or Infusion Indicated AND Responds Quickly to Medications Prophylaxis < 24 Hours  5 (3-6) | **Allergic Reaction**  Prolonged (Not rapidly responsive to medical intervention) AND Recurrence of Symptoms Following Medical Treatment Hospitalization Indicated  6 (5-8) | **Allergic Reaction**  Life Threatening Consequences AND Urgent ICU Level Care Indicated (e.g. Stevens Johnson Syndrome, Anaphylaxis, Angioedema)  10 (9-10) |
| **Sexual Dysfunction** | **Sexual Dysfunction**  Mild Sexual Dysfunction Not Adversely Affecting Relationship  3 (2-4) | **Sexual Dysfunction**  Moderate Sexual Dysfunction AND  Adversely Affecting Relationship  6 (5-7 | **Sexual Dysfunction**  Severe Increase in Sexual Interest Leading to Dangerous Behavior  8 (6-9) |  |
| **Edema** | **Edema Limbs**  Asymptomatic or Mild Symptoms  Clinical or Diagnostic Observations Only  2 (2-4) | **Edema Limbs**  Moderate Symptoms AND  Minimal, Local, or Non-Invasive Intervention Indicated  3 (3-5) | **Edema Limbs**  Severe or Medically Significant but Not Life-Threatening  AND  Hospitalization or Prolongation Existing Hospitalization Indicated  7 (6-8) |  |
| **Fatigue** | **Fatigue**  Fatigue Relieved By Rest  3 (2-4) | **Fatigue**  Fatigue Not Relieved by Rest  3 (2-4) | **Fatigue**  Fatigue Not Relieved by Rest AND Limiting Self Care Activities Daily Living  6 (5-7) |  |
| **Anemia** | **Anemia**  Hemoglobin (Hbg) < Lower Limit Normal - 10 g/dL OR  Hgb < Lower Limits Normal - 6.2mmol/L OR  Hgb < Lower Limits Normal - 100g/L  3 (2-4) | **Anemia**  Hemoglobin (Hbg) < 10- 8 g/dL OR  Hgb < 6.2 - 4.9 mmol/L OR  Hgb < 100 - 80g/L  4 (3-5) | **Anemia**  Hemoglobin (Hbg) < 8 g/dL OR  Hgb < 4.9 mmol/L OR  Hgb < 80 g/L AND Transfusion Indicated  8 (6-8) | **Anemia**  Life-Threatening Consequences AND  Urgent Intervention Indicated  9 (8-10) |
| **DIC** | **Disseminated Intravascular Coagulation**  Lab Findings with No Bleeding  5 (4-7) | **Disseminated Intravascular Coagulation**  Lab Findings with Bleeding  9 (8-10) | **Disseminated Intravascular Coagulation**  Life-Threatening Consequences AND Hospitalization and Urgent Intervention Indicated  10 (9-10) |  |
| **Dyskinesia** | **Dyskinesia**  Mild Restlessness or Increased Motor Activity  4 (3-5) | **Dyskinesia**  Moderate Restlessness or Increased Motor Activity AND Limiting Instrumental Activities Daily Living  7 (6-8) | **Dyskinesia**  Severe Restlessness or Increased Motor Activity AND Limiting Self Care Activities Daily Living  6 (5-8) |  |
| **Kidney Stones** | **Renal Calculi (Kidney Stones)**  Asymptomatic of Mild Symptoms AND  Occasional Use of Non-Prescription Agents  3 (2-4) | **Renal Calculi (Kidney Stones)**  Symptomatic AND Oral Anti-emetics OR Around the Clock Non-Prescription Analgesics or Any Oral Narcotic  6 (4-7) | **Renal Calculi (Kidney Stones)**  Hospitalization Indicated AND IV Intervention Elective Endoscopic or Radiographic Intervention Indicated  7 (6-8) | **Renal Calculi (Kidney Stones)**  Life-Threatening Complications AND Urgent Endoscopic or Operative Intervention and Hospitalization Indicated  9 (8-10) |
| **Insomnia** | **Insomnia**  Mild Difficulty Falling Asleep, Staying Asleep, or Waking Up Early  3 (2-4) | **Insomnia**  Moderate Difficulty Falling Asleep, Staying Asleep, or Waking Up Early  3 (3-5) | **Insomnia**  Severe Difficulty Falling Asleep, Staying Asleep, or Waking Up Early  6 (5-7) |  |
| **Pancreatitis** | **Pancreatitis**  Enzyme Elevation or Radiologic Findings Only  3 (2-5) | **Pancreatitis**  Severe Pain, Vomiting AND Medical Intervention Indicated (e.g. analgesia, nutritional support)  8 (7-9) | **Pancreatitis**  Life-Threatening Consequences AND Hospitalization and Urgent Intervention Indicated  9 (8-10) |  |
| **Flu Reaction** | **Flu Like Symptoms**  Mild Flu-Like Symptoms  2 (2-3) | **Flu Like Symptoms**  Moderate Flu-Like Symptoms > 1 day  2 (2-3) | **Flu Like Symptoms**  Severe Flu-Like Symptoms > 1 Day AND Limiting Self Care Activities Daily Living  5 (4-7) |  |
| **Gait Dysfunction** | **Gait Disturbance**  Mild Change in Gait  (e.g. wide based, limping, or hobbling)  5 (4-6) | **Gait Disturbance**  Moderate Change in Gait (e.g. wide based, limping, or hobbling) AND Assistive Device Indicated  5 (4-7) | **Gait Disturbance**  Severe Change in Gait AND Disabling Requires Wheelchair  9 (8-10) |  |
| **Febrile Neutropenia** | **Febrile Neutropenia**  ANC < 1000/mm3 with Single Temp > 38.3C (101F) OR  Sustained Temp >/= 38C (100.4) for more than 1 Hour  7 (5-8) | **Febrile Neutropenia**  Life-Threatening Consequences AND  Hospitalization and Urgent Intervention Indicated  9 (8-10) |  |  |
|  |  |  |  |  |
| **Laboratory Abnormalities** |  |  |  |  |
| **INR Elevation** | **INR Increase**  INR > 1 - 1.5 x Upper Limit Normal   OR  INR > 1 - 1.5 x Above Baseline if on Anticoagulation  3 (2-4) | **INR Increase**  INR > 1.5 - 2.5 x Upper Limit Normal   OR  INR > 1.5 - 2.5 x Above Baseline if on Anticoagulation  5 (3-7) | **INR Increase**  INR > 2.5 x Upper Limit Normal  OR  INR > 2.5 x Above Baseline if on Anticoagulation  7 (5-7) |  |
| **ALT/AST Elevation** | **ALT or AST Elevation**  Lab 2 - 3 x Upper Limit Normal  3 (2-4) | **ALT or AST Elevation**  Lab 3 - 5 x Upper Limit Normal  4 (3-5) | **ALT or AST Elevation**  Lab 5 -20 x Upper Limit Normal  7 (5-8) | **ALT or AST Elevation**  Lab > 20 x Upper Limit Normal  8 (6-9) |
| **Neutropenia** | **Neutrophil Count Reduced**  ANC < Lower Limit Normal - 1500/mm3 OR  ANC < Lower Limit Normal - 1.5 x 10e9/L  4 (3-6) | **Neutrophil Count Reduced**  ANC < 1500 - 1000/mm3 OR  ANC < 1.5 - 1 x 10e9/L  5 (4-6) | **Neutrophil Count Reduced**  ANC < 1000 - 500/mm3 OR  ANC < 1 - 0.5 x 10e9/L  8(5-8) | **Neutrophil Count Reduced**  ANC < 500/mm3 OR  ANC < 0.5 x 10e9/L  8 (6-9) |
| **Low Platelets** | **Platelet Count Reduced**  Platelets < Lower Limit Normal - 75,000/mm3 OR  Platelets < Lower Limit Normal - 75 x 10e9/L  4 (2-6) | **Platelet Count Reduced**  Platelets < 75,000 - 50,000/mm3 OR  Platelets < 75 - 50 x 10e9/L  7 (5-9) | **Platelet Count Reduced**  Platelets < 50,000 - 25,000/mm3 OR  Platelets < 50 - 25 x 10e9/L  5 (3-6.5) | **Platelet Count Reduced**  Platelets < 25,000/mm3 OR  Platelets < 25 x 10e9/L  7 (5-9) |
| **Hypernatremia** | **Hypernatremia**  Na > Upper Limit Normal - 150 mmol/L  4 (2-5) | **Hypernatremia**  Na > 150 - 155 mmol/L  4 (3-5) | **Hypernatremia**  Na > 155 - 160 mmol/L AND   Hospitalization Indicated  8 (6-9) | **Hypernatremia**  Na > 160 mmol/L and Life Threatening Consequences  Hospitalization Indicated  8 (8-9) |
| **Hyponatremia** | **Hyponatremia**  Na < 130 mmol/L - Lower Limit Normal  3 (2-4) | **Hyponatremia**  Na < 120 - 130 mmol/L  5 (4-6) | **Hyponatremia**  Na < 120 mmol/L  Life Threatening Complications  9 (7-9) |  |
| **Hyperkalemia** | **Hypokalemia**  K < 3 mmol/L - Lower Limit Normal  4 (2-6) | **Hypokalemia**  K < 3 mmol/L - Lower Limit Normal  AND Symptomatic Intervention Indicated  6 (5-8) | **Hypokalemia**  K < 2.5 - 3 mmol/L  AND  Hospitalization Indicated  6 (6-8) | **Hypokalemia**  K < 2.5 mmol/L with Life Threatening Consequences AND Urgent Hospitalization  9 (7-10) |
| **Hypokalemia** | **Hyperkalemia**  K > Upper Limits Normal - 5.5 mmol/L  3 (2-4) | **Hyperkalemia**  K > 5.5 - 6 mmol/L  6 (5-7.5) | **Hyperkalemia**  K > 6 - 7 mmol/L  6 (4-7) | **Hyperkalemia**  K > 7 mmol/L  9 (8-10) |
| **Urine Retention** | **Urinary Retention**  Urinary or Suprapubic Catheter Not Indicated  Able to Void with Some Residual Volume  4 (3-5) | **Urinary Retention**  Placement of Urinary, Suprapubic, or Intermittent Catheter Placement Indicated AND Medication Indicated  6 (5-6) | **Urinary Retention**  Elective Operative or Radiologic Intervention Indicated AND Substantial Loss of Kidney Function or Mass  8 (8-10) | **Urinary Retention**  Life-Threatening Consequences AND Organ Failure Urgent Operative Intervention Indicated  9 (8-10) |

**Appendix 2: Sample Adult and Pediatric Physician Surveys**

| **Adverse Event Unit Pediatric Form** | Resize font:  [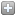](javascript:;)\|[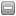](javascript:;) |
| --- | --- |

**Project Objectives:**

The objective of this study is to assign value to adverse events (AE's) independent of any particular disease or medication. The results of this study will be used to create a common unit that can be used to compare the significance of treatment associated adverse events. Adverse events and terminology are adapted from the Common Terminology Criteria for Adverse Events (CTCAE) version 4.

This project is sponsored by the 2016 American Academy of Neurology, American Brain Foundation, and Myasthenia Gravis Foundation of America Clinician Scientist Development Award.

**Project Instructions:**

You are evaluating a small subset of the adverse events being studied in this project. You will not see all the possible AE’s within a particular category (e.g. you may evaluate only 1 of 5 possible AE’s in the category of hypertension). Other subjects are evaluating the other AE’s within these categories. Consider the scores you assign as you rate the AE’s across different categories. **Please consider these adverse events independent of any particular disease or medication.**

**Below are a series of adverse events that may be the result of any medical treatment. Please assign a score from 0 (no significance) to 10 (most significant) to each adverse event below. Using your medical expertise, please consider the duration and long-term sequelae of each AE as you assign values.**

This survey should take about 10 minutes to complete.

Thank you for participating.

|  | **Are you Board Certified or Board Eligible in your medical specialty?**  * must provide value | Yes  No  [reset](javascript:;) |
| --- | --- | --- |
| **Hypertension Caused By Medical Intervention** | | |
|  | **Pre-Hypertension**  **Systolic Blood Pressure: 120-139 or**  **Diastolic Blood Pressure: 80-89**  * must provide value | 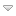 |
|  | **Hypertension**  **Systolic Blood Pressure: > 160 or**  **Diastolic Blood Pressure: > 100**  * must provide value | 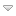 |
|  | **Life Threatening Hypertension**  **Hospitalization for Hypertensive Urgency/Emergency**  * must provide value | 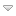 |
| **Depression Caused by Medication** | | |
|  | **Depression**  **Moderate Depressive Symptoms AND**  **Limiting Instrumental ADL**  * must provide value | 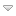 |
|  | **Depression**  **Life-threatening consequences**  **Threats of Harm to Self or Others**  **Hospitalization Indicated**  * must provide value | 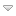 |
| **Headache Caused By Medical Intervention** | | |
|  | **Headache**  **Mild Pain < 1 week**  * must provide value | 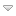 |
|  | **Headache**  **Severe Pain**  **Limits Self Care and Activities Daily Living < 1 week**  * must provide value | 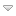 |
|  | **Chronic Headache**  **Moderate Pain or More**  **Duration > 1 week**  * must provide value | 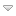 |
| **Neural Tube Defects Caused By Medical Intervention** | | |
|  | **Myelomenigocele:**  **Cleft in vertebral column**  * must provide value | 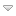 |
|  | **Anencephaly:**  **Open deficit, cranial tube exposed. Not compatible with survival.**  * must provide value | 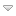 |
| **Teratogenic Cognitive Dysfunction**  **Caused By Medical Intervention** | | |
|  | **Mild Intellectual Disability:**  **Children require academic supports to learn skills appropriate for age. Social skills and personal judgment immature for age. Most individuals independent in daily living activities, employable in jobs, and able to live independently.**  * must provide value | 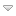 |
|  | **Severe Intellectual Disability:**  **Little understanding of written language, numbers, time, money concepts. Caretakers provide extensive support. Benefit from interaction with family/familiar people with limited social interaction. Trainable in some basic activities of daily living.**  * must provide value | 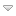 |
| **Hallucinations Caused By Medical Intervention** | | |
|  | **Hallucinations**  **Severe Hallucinations**  **Medical Intervention Indicated**  **Hospitalization Not Indicated**  * must provide value | 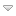 |
|  | **Hallucinations**  **Life-threatening Complications**  **Threats of Harm to Self or Others**  **Hospitalization Indicated**  * must provide value | 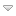 |
| **Dry Mouth Caused By Medical Intervention** | | |
|  | **Dry Mouth**  **Symptomatic (e.g. dry thick saliva)**  **Without Significant Dietary Alteration**  * must provide value | 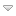 |
| **Gastric or Duodenal Ulcer Caused By Medical Intervention** | | |
|  | **Gastric or Duodenal Ulcer**  **Symptomatic**  **Altered GI Function**  **Medical Intervention Indicated**  * must provide value | 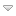 |
|  | **Gastric or Duodenal Ulcer**  **Severely Altered GI Function**  **TPN Indicated OR**  **Elective Operative or Endoscopic Intervention Indicated**  * must provide value | 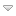 |
| **Cushingoid Features Caused By Medical Intervention** | | |
|  | **Cushingoid**  **Mild Symptoms**  **Intervention Not Indicated**  * must provide value | 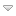 |
|  | **Cushingoid**  **Severe Symptoms**  **Medical Intervention or Hospitalization Indicated**  * must provide value | 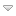 |
| **Endocrine Disorders Caused By Medical Intervention** | | |
|  | **Endocrine Disorders**  **Moderate Symptoms**  **Minimal, Local, or Non-invasive Intervention Only**  * must provide value | 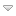 |
|  | **Endocrine Disorders**  **Life-threatening Consequences**  **Urgent Intervention Indicated**  * must provide value | 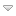 |
| **Neutrophil Count Reduced Due to Medical Intervention** | | |
|  | **Neutrophil Count Reduced**  **ANC < Lower Limit Normal - 1500/mm3 OR**  **ANC < Lower Limit Normal - 1.5 x 10e9/L**  * must provide value | 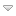 |
|  | **Neutrophil Count Reduced**  **ANC < 1000 - 500/mm3 OR**  **ANC < 1 - 0.5 x 10e9/L**  * must provide value | 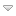 |
| **Drug Related Myalgia** | | |
|  | **Drug Related Myalgia**  **Mild Pain**  * must provide value | 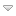 |
|  | **Drug Related Myalgia**  **Moderate Pain**  **Limiting Instrumental Activities Daily Living**  * must provide value | 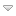 |
| **Congenital Malformations Due to Medical Intervention** | | |
|  | **Intrauterine Growth Restriction:**  **Birth weight below 10th percentile for gestational age. Requires additional monitoring. May have clinical consequences.**  * must provide value | 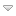 |
|  | **Genetic Disorders:**  **Genetic disorders compatible with survival but likely to result in disability.**  **(e.g. Chromosomal Disorders, Trisomy 21)**  * must provide value | 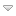 |
|  | **Microcephaly/Macrocephaly:**  **Abnormalities in head circumference and brain size. Requires additional monitoring. May result in cognitive dysfunction.**  * must provide value | 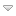 |
| **Pruritus (Itching) Caused By Medical Intervention** | | |
|  | **Pruritus (Itching)**  **Intense or Widespread Intermittent**  **Skin Changes from Scratching**  **Oral Intervention Indicated**  * must provide value | 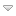 |
|  | **Pruritus (Itching)**  **Intense or Widespread, Constant**  **Oral Corticosteroid or Immunosuppressive Therapy Indicated**  * must provide value | 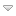 |
|  | **Name**  **Please list your name if you would like to receive acknowledgement when published.** |  |
|  | **Email**  **Please list a current email address** |  |
|  | **Medical Specialty**  **Please list your medical specialty with highest degree of subspecialization**  **(e.g. neuromuscular neurology)**  * must provide value |  |
|  | **Years of Practice**  **Please list years of clinical practice after completion of highest degree of training (e.g. residency, fellowship)**  * must provide value |  |
|  | **Do you practice in an academic practice?**  * must provide value | Yes  No  [reset](javascript:;) |
|  | **Location of Practice**  **Please list state of practice.**  **Please also list country if outside the United States**  * must provide value |  |
|  | **Sex**  * must provide value | 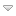 |
| \| Submit \| \| --- \| | | |

[Powered by REDCap](https://projectredcap.org/)

| **Adverse Event Unit Adult Form** | Resize font:  [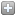](javascript:;)\|[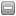](javascript:;) | [**Returning?**](https://redcap.med.uvm.edu/surveys/?s=PLD8FDHMR4&__return=1) |
| --- | --- | --- |

**Project Objectives:**

The objective of this study is to assign value to adverse events (AE's) independent of any particular disease or medication. The results of this study will be used to create a common unit that can be used to compare the significance of treatment associated adverse events. Adverse events and terminology are adapted from the Common Terminology Criteria for Adverse Events (CTCAE) version 4.

This project is sponsored by the 2016 American Academy of Neurology, American Brain Foundation, and Myasthenia Gravis Foundation of America Clinician Scientist Development Award.

**Project Instructions:**

You are evaluating a small subset of the adverse events being studied in this project. You will not see all the possible AE’s within a particular category (e.g. you may evaluate only 1 of 5 possible AE’s in the category of hypertension). Other subjects are evaluating the other AE’s within these categories. Consider the scores you assign as you rate the AE’s across different categories. **Please consider these adverse events independent of any particular disease or medication.**

**Below are a series of adverse events that may be the result of any medical treatment. Please assign a score from 0 (no significance) to 10 (most significant) to each adverse event below. Using your medical expertise, please consider the duration and long-term sequelae of each AE as you assign values.**

This survey should take about 10 minutes to complete.

Thank you for participating.

|  | **Are you Board Certified or Board Eligible in your medical specialty?**  * must provide value | Yes  No  [reset](javascript:;) |
| --- | --- | --- |
| **Hypertension Caused by Medical Intervention** | | |
|  | **Hypertension**  **Systolic Blood Pressure: 140-159 or**  **Diastolic Blood Pressure: 90-99**  **OR**  **Increase or Start Anti-hypertension medications**  * must provide value | 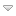 |
|  | **Life Threatening Hypertension**  **Hospitalization for Hypertensive Urgency/Emergency**  * must provide value | 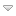 |
| **Diabetes Caused by Medical Intervention** | | |
|  | **Pre-Diabetes**  **Hemoglobin A1C: 5.7 - 6.4% or**  **Fasting Glucose: 100 - 125mg/dL or**  **2H Oral Gluc Tolerance Test: 140 - 199 mg/dL**  * must provide value | 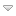 |
|  | **Acute Life Threatening Diabetes**  **Hospitalization (e.g. Ketoacidosis)**  * must provide value | 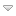 |
| **Headache Caused by Medical Intervention** | | |
|  | **Headache**  **Moderate Pain**  **AND**  **Limits Activities Daily Living < 1 week**  * must provide value | 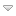 |
|  | **Chronic Headache**  **Moderate Pain or More**  **AND**  **Duration > 1 week**  * must provide value | 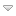 |
| **Suicidal Ideation Caused by Medical Intervention** | | |
|  | **Suicidal Ideation**  **Increased Thoughts of Death But No Wish to Kill Oneself**  * must provide value | 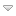 |
|  | **Suicidal Ideation**  **Specific Plan to Commit Suicide OR**  **Suicide Attempt without Serious Intent to Die**  **May Not Require Hospitalization**  * must provide value | 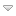 |
| **Teratogenic Cognitive Dysfunction**  **Caused by Medical Intervention** | | |
|  | **Teratogenic Moderate Intellectual Disability:**  **Conceptual and academic skills lag well behind peers. Adults able attain elementary level skills. Social cues, judgment, and life decisions require support. Most capable of personal care with support (e.g. group home).**  * must provide value | 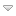 |
|  | **Teratogenic Severe Intellectual Disability:**  **Little understanding of written language, numbers, time, money concepts. Caretakers provide extensive support. Benefit from interaction with family/familiar people with limited social interaction. Trainable in some basic activities of daily living.**  * must provide value | 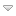 |
| **Hallucinations Caused by Medical Intervention** | | |
|  | **Hallucinations**  **Mild Hallucinations (e.g. perceptual distortions)**  * must provide value | 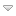 |
|  | **Hallucinations**  **Moderate Hallucinations**  * must provide value | 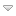 |
| **Dry Mouth Caused by Medical Intervention** | | |
|  | **Dry Mouth**  **Moderate Symptoms**  **AND**  **Oral Intake Alterations**  **(e.g. copious water, other lubricants) OR**  **Diet Limited to Purees**  * must provide value | 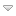 |
|  | **Dry Mouth**  **Inadequate Oral Intake**  **AND**  **Tube Feeds, TPN Indicated**  * must provide value | 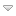 |
| **Gastric or Duodenal Ulcer Caused by Medical Intervention** | | |
|  | **Gastric or Duodenal Ulcer**  **Asymptomatic**  **AND**  **Diagnostic Observations Only**  **Intervention Not Indicated**  * must provide value | 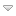 |
|  | **Gastric or Duodenal Ulcer**  **Life-threatening Complications**  **AND**  **Urgent Operative Intervention Indicated**  * must provide value | 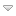 |
| **Cushingoid Features Caused by Medical Intervention** | | |
|  | **Cushingoid**  **Moderate Symptoms AND**  **Medical Intervention Indicated**  * must provide value | 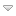 |
| **Endocrine Disorders Caused by Medical Intervention** | | |
|  | **Endocrine Disorders**  **Asymptomatic or Mild Symptoms AND**  **Clinical or Diagnostic Observations Only**  * must provide value | 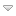 |
|  | **Endocrine Disorders**  **Severe or Medically Significant but Not Immediately Life-threatening**  **AND**  **Hospitalization or Prolongation of Existing Hospitalization**  * must provide value | 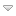 |
| **Neutrophil Count Reduced Caused by Medical Intervention** | | |
|  | **Neutrophil Count Reduced**  **ANC < 1500 - 1000/mm3 OR**  **ANC < 1.5 - 1 x 10e9/L**  * must provide value | 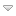 |
|  | **Neutrophil Count Reduced**  **ANC < 500/mm3 OR**  **ANC < 0.5 x 10e9/L**  * must provide value | 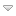 |
| **Platelet Count Reduced Due To Medical Intervention** | | |
|  | **Platelet Count Reduced**  **Platelets < Lower Limit Normal - 75,000/mm3 OR**  **Platelets < Lower Limit Normal - 75 x 10e9/L**  * must provide value | 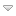 |
|  | **Platelet Count Reduced**  **Platelets < 75,000 - 50,000/mm3 OR**  **Platelets < 75 - 50 x 10e9/L**  * must provide value | 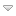 |
| **Drug Related Myalgia Caused by Medical Intervention** | | |
|  | **Drug Related Myalgia**  **Severe Pain AND**  **Limiting Self Care Activities Daily Living**  * must provide value | 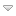 |
| **Secondary Malignancy Caused by Medical Intervention** | | |
|  | **Treatment Related Secondary Malignancy**  **Non-Life Threatening Secondary Malignancy**  * must provide value | 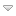 |
|  | **Treatment Related Secondary Malignancy**  **Acute Life Threatening Secondary Malignancy**  **(e.g. blast crisis)**  * must provide value | 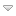 |
| **Pruritus (Itching) Caused by Medical Intervention** | | |
|  | **Pruritus (Itching)**  **Mild or Localized AND**  **Topical Intervention Indicated**  * must provide value | 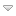 |
| **Skin Disorders Caused by Medical Intervention** | | |
|  | **Skin Disorders**  **Moderate, Minimal, Local, or Non-Invasive Intervention Indicated**  * must provide value | 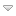 |
|  | **Skin Disorders**  **Severe or Medically Significant but Not Immediately Life Threatening**  **AND**  **Hospitalization or Prolongation of Existing Hospitalization**  * must provide value | 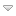 |
| **Please provide the following demographic information.**  **If you provide us your name and email, you will be acknowledged at the time of publication.** | | |
|  | **Name**  **Please list your name if you would like to receive acknowledgement when published.** |  |
|  | **Email**  **Please list a current email address** |  |
|  | **Medical Specialty**  **Please list your medical specialty with highest degree of subspecialization**  **(e.g. neuromuscular neurology)**  * must provide value |  |
|  | **Years of Practice**  **Please list years of clinical practice after completion of highest degree of training (e.g. residency, fellowship)**  * must provide value |  |
|  | **Do you practice in an academic practice?**  * must provide value | Yes  No  [reset](javascript:;) |
|  | **Location of Practice**  **Please list state of practice.**  **Please also list country if outside the United States**  * must provide value |  |
|  | **Sex**  * must provide value | 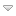 |

**Appendix 3: All Items Included in the Potential Patient Discrete Choice**

**Diabetes:**

Diabetes mellitus refers to a group of diseases that affect how your body uses blood sugar (glucose). Too much glucose can lead to serious health problems. Depending on what type of diabetes you have, blood sugar monitoring, injectable insulin and oral medications may play a role in your treatment

Complication: (AEU 6)

You have developed Type 2 Diabetes as the result of a medical treatment. Discontinuing the medication may not be enough to reverse this condition. The severity of diabetes requires treatment with multiple oral or injected medications. Long-term diabetes at this level increases the risk of: cardiovascular disease (heart attack/stroke), nerve damage (numbness), kidney damage, eye damage, and other complications. Treatment may reduce the risk of these complications.

**Osteoporosis:**

Osteoporosis causes bones to become weak and brittle. Bone fractures, particularly in the spine or hip, are the most serious complication of osteoporosis. Treatment may include exercise, vitamin supplements, oral medications, and in severe cases injectable treatments.

Complication: (AEU 4)

You have developed Osteopenia as a result of a medical treatment. Osteopenia is bone loss that is **NOT severe enough** to meet the diagnosis of osteoporosis. Patients with osteopenia are at risk to develop osteoporosis in the future. This may be a reversible condition with discontinuing medication. At this level, no medical therapies are recommended and no complications of osteoporosis will develop.

**Weight Gain:**

Weight gain can result in moving from a status of normal weight to overweight or obese. Obesity isn't just a cosmetic concern. Developing obesity increases your risk of diseases and health problems, such as heart disease, diabetes and high blood pressure. Discontinuation of medicine may not be enough to lose gained weight.

Complication: (AEU 6)

You have developed more than a 20% increase (More than 36 pounds for a 180 pound person) in your weight as a result of medical treatment. This amount of weight gain is enough to move from a status of normal weight to overweight, or from overweight to obese. Long-term increased weight increases your risk of diseases and health problems, such as heart disease, diabetes and high blood pressure. This degree of weight gain may not improve with stopping the medication alone.

**Cognitive Impairment:**

Cognitive impairment can involve problems with memory, language, thinking and judgment that are greater than normal age-related changes. Drug induced cognitive dysfunction is likely reversible if the offending medication is stopped.

Complication: (AEU 4)

You have developed mild cognitive impairment as a result of medical treatment. The symptoms are mild and do not interfere with work, school, or life performance. This condition is likely reversible if the medication is stopped.

**Seizure:**

A generalized seizure features a loss of consciousness and violent muscle contractions. Seizures are caused by abnormal electrical activity throughout the brain. Short-lived seizures do not result in injury to the brain. Prolonged seizures that last for 30 or more minutes may result in some changes to the brain. Patients who develop repeated seizures often require medical treatment to prevent additional events. Patients who experience a drug induced seizure are unlikely to experience another one if the medication is stopped.

Complication: (AEU 6)

You experience a short, generalized seizure with loss of consciousness as a result of medical treatment. This short seizure does not result in injury to the brain and does not require admission to a hospital. It is unlikely to occur again if the offending medication is stopped. Your doctor may recommend avoiding activities such as driving or swimming/bathing alone, for a few months following the event in case you experienced a second loss of consciousness. Your doctor is unlikely to recommend treatment with an anti-seizure medication.

**Heart Attack:**

A heart attack occurs when the flow of blood to the heart is blocked. The interrupted blood flow can damage or destroy part of the heart muscle. These conditions require emergency medical care. In severe cases patients require ICU (Intensive Care Unit) level care. Patients typically require a combination of medical and surgical treatments. These conditions increase risk for abnormal heart rhythms, heart failure, and in severe cases death.

Complication: (AEU 9)

You develop a heart attack as the result of medical treatment. This heart attack requires medical and surgical interventions. It also requires care in an Intensive Care Unit (ICU) because of ongoing heart dysfunction and the risk for developing an abnormal heart rhythm that could result in death. Long-term heart dysfunction is likely after the acute hospitalization.

**Deep Vein Thrombosis (DVT):**

Deep vein thrombosis (DVT) occurs when a blood clot forms in one or more of the deep veins in your body, usually in your legs. Deep vein thrombosis can cause leg pain or swelling, but also can occur with no symptoms. Deep vein thrombosis can be very serious because blood clots in your veins can break loose and lodge in your lungs, blocking blood flow (pulmonary embolism). Treatment of DVT includes anticoagulant medications (blood thinners) and in severe circumstances, placement of a filter in your blood vessels or treatment with a clot busting medication. Blood thinner treatment increases the risk for bleeding.

Complication: (AEU 7)

You have developed a DVT in your leg as a result of medical treatment. No complications have occurred with this DVT, such as pulmonary embolism. You require treatment with a blood thinner for at least a few months. You may have been admitted to the hospital for a short time due to this issue.

**Headache**Headaches may include syndromes that cause discomfort on the head including throbbing pain, stabbing pain, and numbness. Severe headaches may result in impaired physical and cognitive function. Severe headaches can impair daily function and may require treatment with medications. Drug induced headaches are likely to improve with stopping an offending medication.

Complication: (AEU 5)

You have developed a severe headache that limits routine daily activities and self-care as a result of medical therapy. This headache lasts less than 1 week, may require a short course of pain medication (such as ibuprofen), and improves with discontinuing the offending medication. No ongoing medical therapy is required.

**Hallucinations**Hallucinations are the symptom of seeing or hearing things that don't exist. For the person experiencing this symptom, the hallucinations have the full force and impact of a normal experience. Hallucinations can be in any of the senses, but hearing voices is the most common hallucination. This symptom can impact the ability to function in day to day life. In severe cases, patients who develop this symptom may require treatment with anti-psychotic medications and psychotherapy. Medication induced hallucinations are likely to improve with stopping the offending medication.

Complication: (AEU 6)

You have developed severe hallucinations as a result of medical therapy. The symptoms are severe enough to affect daily life activities and to require treatment with anti-psychotic medications. The symptoms are not severe enough to require admission to the hospital. Symptoms are likely to improve if offending medication is stopped.

**Treatment Related Malignancy (Cancer)**

Cancer refers to any one of a large number of diseases characterized by the development of abnormal cells that divide uncontrollably and have the ability to infiltrate and destroy normal body tissue. Cancer often has the ability to spread throughout your body. Cancer is the second-leading cause of death in the United States. Treatment of cancer may include surgery, chemotherapy, radiation therapy, hormone therapy, and immune system therapy. Cancer that develops as an adverse complication of a medical treatment will not improve with removal of the offending medication.

Complication: AEU 9

You develop cancer as a complication of medical therapy. The cancer is metastatic and is likely to shorten your life expectancy. Treatment with some combination of surgery, chemotherapy, radiation therapy, immune system therapy, and hormone therapy is likely indicated depending on the type of cancer. Stopping the offending medication will not result in cure of the cancer.

**Myalgia (Muscle Pain):**

Myalgias are deep muscle aches. When severe, pain may impair daily function. Treatment with pain medications may relieve the symptom. Myalgia caused as an adverse effect of medical treatment will likely improve when the offending medication is stopped.

Complication: AEU 6

You develop severe myalgia (muscle pain) as a result of medical therapy. This severe pain limits routine daily activities and self-care. You may require pain medication to help relieve the symptoms. The pain is likely to improve with stopping the offending medication.

**Itchy Skin (Pruritis)**

Itchy skin is an uncomfortable, irritating sensation that makes you want to scratch. Repeated scratching can cause raised, thickened areas of skin that may bleed or become infected. Itchy skin may require treatment with topical creams or oral medications. The itchy skin will likely improve when the offending medication is stopped.

Complication: AEU 6

You develop intense, widespread, constant itching as a result of medical therapy. This severe itching limits routine daily activities and self-care. You may require oral immune system medications in addition to topical creams to help relieve the symptoms. The itching is likely to improve with stopping the offending medication.

**Low Platelets:**

Platelets are colorless blood cells that help blood clot. Platelets stop bleeding by clumping and forming plugs in blood vessel injuries. Low platelets may cause few signs or symptoms. In rare cases, the number of platelets may be so low that dangerous external or internal bleeding can occur.

Complication: (AEU 5):

You develop a moderate level of low platelets as a result of medical therapy. At this platelet level, you may be at risk for excessive bleeding during a surgical procedure or with severe injury. It is NOT likely that you would experience excessive bleeding with minor injury. Your platelet level is likely to return to normal if the offending medication is stopped.

**Low Blood Sodium (Hyponatremia):**

Hyponatremia is a condition that occurs when the level of sodium in your blood is abnormally low. Sodium is an electrolyte, and it helps regulate the amount of water that's in and around your cells. When this happens, your body's water levels rise, and your cells begin to swell. This swelling can cause many health problems, from mild to life-threatening. Depending on the cause of low blood sodium, you may simply need to cut back on how much you drink. In other cases of low blood sodium, you may need intravenous fluids and medications.

Complication: (AEU 9)

You develop low blood sodium as a result of medical therapy. The blood sodium level is low enough to be life threatening. You will require admission to the hospital to correct the level and administration of IV or oral medications. This condition is likely reversible with appropriate medical treatment and discontinuation of the offending medication.

**Flu-Like Symptoms:**

Patients can develop muscle aches, fever, chills, and other symptoms of the flu without infection with the flu virus following administration of some medications. These symptoms may be treatable with anti-inflammatory medications. Discontinuing the offending medication will typically result in resolution of these symptoms.

Complication: (AEU 2)

You develop mild, flu-like symptoms as the result of medical therapy. These symptoms do not impact daily function. The symptoms are likely to improve with treatment with a non-steroidal medication (Advil/Ibuprofen) or acetaminophen (Tylenol). The symptoms will also improve with discontinuation of the offending medication.

**Abnormal Movements (Dyskinesia):**

Abnormal, involuntary movements of limbs or head can develop as a result of medical therapies. These movements can include movements of the tongue, lip puckering, movement of arms, foot tapping, shoulder shrugging. The movements vary from unnoticeable to others to severe, violent movements. The movements may or may not be reversible when offending medication is stopped.

Complication: (AEU 4)

You develop mild restlessness or mild increased limb movements as a result of medical therapy. These symptoms do not impact daily function. They may be noticeable to others. The symptoms may improve with discontinuing the offending medication.

**Kidney Stones:**

Kidney stones are hard deposits made of minerals and salts that form inside your kidneys. Passing kidney stones can be quite painful, but the stones usually cause no permanent damage if they're recognized in a timely fashion. Depending on your situation, you may need nothing more than to take pain medication and drink lots of water to pass a kidney stone. In other instances — for example, if stones become lodged in the urinary tract, are associated with a urinary infection or cause complications — surgery may be needed.

Complication: (AEU 7)

You develop kidney stones as a result of medical therapy. The kidney stones are severe enough that hospitalization is needed. Treatment with IV medications and radiological (ultrasound) procedures are likely needed. Recovery is expected with appropriate treatment.

**Diarrhea:**

Diarrhea is defined as loose, watery and possibly more-frequent bowel movements. In most cases, diarrhea lasts a couple of days. But when diarrhea lasts for weeks, it can indicate a serious disorder, result in dehydration, and may require hospitalization.

Complication: (AEU 4)

You develop diarrhea as the result of medical therapy. You are experiencing 4 to 6 stools per day more than typical for you. At this level, the diarrhea may be impacting daily function. Diarrhea will likely improve with discontinuing the offending medication.

**Pulmonary Fibrosis**

Pulmonary fibrosis is a lung disease that occurs when lung tissue becomes damaged and scarred. This thickened, stiff tissue makes it more difficult for your lungs to work properly. As pulmonary fibrosis worsens, you become progressively more short of breath. The lung damage caused by pulmonary fibrosis can't be repaired. Complications from this condition can shorten life expectancy. Medications and therapies can sometimes help ease symptoms and improve quality of life. For some people, a lung transplant might be appropriate.

Complication: (AEU 9)

You develop pulmonary fibrosis as a result of medical therapy. Tissue damage is discovered in 75% of your lung tissue. You develop severe shortness of breath at rest due to low oxygen levels. You have also developed the complication of congestive heart failure due to the pulmonary fibrosis. This level of pulmonary fibrosis will shorten life expectancy and will not improve with stopping the offending medication.

**Hypertension (High Blood Pressure)**

High blood pressure is a common condition in which the pressure against your artery walls is high enough that it may eventually cause health problems, such as heart disease. Even without symptoms, damage to blood vessels and your heart continues and can be detected. Uncontrolled high blood pressure increases your risk of serious health problems, including heart attack and stroke. High blood pressure caused by a medication side effect may or may not improve with removing the offending medication.

Complication: (AEU 4)

You develop high blood pressure as a result of medical therapy. Your high blood pressure is considered to be at a mild to moderate level. This level of high blood pressure may require treatment with a medication to lower blood pressure. Long exposure to this level of high blood pressure raises the risk of cardiovascular complications (for example heart attack). Blood pressure is likely to return to normal with stopping the offending medication or starting treatment for high blood pressure.

**Liver Disease**

The liver is an organ in your abdomen that is essential for digesting food and ridding your body of toxic substances. Damage to the liver results in scarring (cirrhosis), which can lead to liver failure. Liver failure can result in complications such as excessive bleeding, confusion, disorientation, nausea/vomiting, abdominal pain and fatigue. Medication induced liver failure may be reversible with removal of the offending medication. In severe cases, liver transplant may be required.

Complication: (AEU 3)

You develop asymptomatic, mild liver failure as a result of medical therapy. At this mild stage, diagnostic procedures (for example CT scan, blood work) may be recommended. You will require observation by your doctor. No short or long-term complications are expected. The liver failure is likely to improve with removal of the offending medication.

**Kidney Failure**

Kidney failure, describes the loss of kidney function. Your kidneys filter wastes and excess fluids from your blood. When chronic kidney disease reaches an advanced stage, dangerous levels of fluid, electrolytes and wastes can build up in your body. Complications of kidney failure may include limb swelling, heart disease, cognitive changes, fatigue, low red blood cells, increased infections, difficult to control high blood pressure. In severe cases, patients may require a kidney transplant. Kidney failure caused by medication side effect may improve with stopping the offending medication.

Complication: (AEU 8)

You develop severe kidney failure as a result of medical therapy. At this level of kidney failure, you will require admission to the hospital with multiple medical interventions. You will experience symptoms including fatigue, limb swelling, high blood pressure, shortness of breath. Stopping the offending medication may or may not result in improvement in kidney failure.

**Infection**

Infectious diseases are disorders caused by bacteria, viruses, fungi, or parasites. Signs and symptoms vary depending on the organism causing the infection, but often include fever and fatigue. Mild infections may respond to rest and medications taken at home, while some life-threatening infections may require hospitalization, treatment with intravenous medications, and supportive care in an intensive care unit (ICU).

Complication: (AEU 8)

You develop a severe infection as a result of medical therapy. The infection requires admission to the hospital and treatment with intravenous medications. The infection is not immediately life threatening but will likely require multiple days in the hospital for you to recover.

**Weight Loss**

Involuntary weight loss can occur as a result of medical therapy. Weight loss of more than 20% (36 pounds for a 180 pound person) of body weight can result in malnutrition, nutrient deficiencies, and organ failure. Mild to moderate weight loss may require patients to consume dietary supplements (for example high calorie shakes). Patients with severe weight loss may require hospital admission, surgical placement of a feeding tube, and administration of liquid food through the feeding tube. Weight loss may stabilize or improve with stopping the offending medication.

Complication: (AEU 8)

You develop severe weight loss of more than 20% (36 pounds for a 180 pound person) of your body mass as a result of medical therapy. The weight loss is so severe that you require admission to the hospital, surgical placement of a feeding tube, and administration of liquid food through the feeding tube. The weight loss is likely to improve with nutrition through the feeding tube and stopping the offending medication.

**Dizziness (Vertigo)**

Dizziness that creates the false sense that you or your surroundings are spinning or moving is called vertigo. Frequent dizzy spells or constant dizziness can significantly affect your life. But dizziness rarely signals a life-threatening condition. Dizziness may be triggered or worsened by walking, standing up or moving your head. Dizziness may accompanied by nausea or be so sudden or severe that you need to sit or lie down. Episodes can last seconds or days and may recur. Dizziness that develops as a complication of medical therapy is likely to improve with stopping the offending medication.

Complication: (AEU 3)

You develop mild, intermittent, short lived, dizziness as a result of medical therapy. The symptoms can be bothersome or mildly uncomfortable but DO NOT impact your ability to function in your normal routine. No medical treatment is recommended. The dizziness will likely improve with stopping the offending medication.

**Hair Loss:**

Hair loss can affect just your scalp or your entire body. Hair loss caused by a medication may result in a gradual or sudden loss of scalp and body hair. The hair loss can range from mild to severe. While the hair loss is not medically dangerous, it can be associated with psychological or social complications. Hair loss associated with a medical treatment may improve over time with stopping the offending medication.

Complication: (AEU 5)

You develop loss of more than 50% of your body hair as a result of a medical therapy. This degree of hair loss will be noticeable to others. In some people this degree of hair loss may result in psychological or social complications. Some patients may use a wig or head cover with this degree of hair loss. Your hair may regrow over time with stopping the offending medication.

**Headache**Headaches may include syndromes that cause discomfort on the head including throbbing pain, stabbing pain, and numbness. Severe headaches may result in impaired physical and cognitive function. Severe headaches can impair daily function and may require treatment with medications. Drug induced headaches are likely to improve with stopping an offending medication.

Complication: (AEU 2)

You have developed a mild headache as a result of medical therapy that lasts less than 1 week and does not impair your ability to function. It may or may not require a short course of pain medication (such as ibuprofen). No ongoing medical therapy is required.

**Congestive Heart Failure**

Heart failure occurs when your heart muscle doesn't pump blood as well as it should. The symptoms of heart failure may include: Shortness of breath, fatigue, swelling of legs, trouble exercising, weight gain, difficulty concentrating. If heart failure worsens, damage to the kidneys, liver, and heart muscle can occur. Treatment of heart failure may include blood pressure medications, placement of a pacemaker or implantable defibrillator in a patient’s chest, and in severe cases heart transplantation. This complication may not completely resolve with removal of the offending medication.

Complication: (AEU 5)

You develop asymptomatic heart failure detected only by laboratory testing or imaging of your heart, as a result of medical therapy. This condition has the potential to develop into more severe heart failure. This condition may or may not improve with stopping the offending medication.

**Stroke:**

A stroke occurs when the blood supply to part of your brain is interrupted or severely reduced, depriving brain tissue of oxygen and nutrients. Within minutes, brain cells begin to die. As a result of the stroke, patients can develop paralysis of arms or legs, trouble talking and swallowing, sensory loss in the limbs, thinking dysfunction. When deficits are severe, some patients must be fed through a tube in the stomach and may need to have 24 hour care at home or in a nursing home following a stay in the hospital.

Complication: (AEU 10)

You develop a severe stroke as a result of medical therapy. The severity of weakness and deficits requires a prolonged hospitalization. Due to the degree of dysfunction, you may require a feeding tube to be placed in the stomach to maintain nutrition and may have trouble communicating. If you survive hospitalization, you will require 24 hour supervised care in a long-term facility, like nursing home.

**Cataracts:**

A cataract is a clouding of the normally clear lens of your eye. Clouded vision caused by cataracts can make it more difficult to read, drive a car (especially at night) or see the expression on a friend's face. Most cataracts develop slowly and don't disturb your eyesight early on. But with time, cataracts will eventually interfere with your vision. Most cataracts can be repaired with a generally safe and effective surgical procedure.

Complication: (AEU 5)

You develop symptomatic cataracts as a result of medical therapy. The cataracts result in a mild to moderate decrease in your vision that may require corrective lenses. A surgery is not required for this level of cataracts. Symptoms may improve or stop getting worse with removal of the offending medication.

**Dry Eyes:**

Dry eyes are a common condition that occurs when your tears aren't able to provide adequate lubrication for your eyes. If you have dry eyes, your eyes may sting or burn. When severe, dry eyes could result in damage to the surface of the eye and decreased quality of life. Treatment with eye drops will usually result in improvement of symptoms. In severe cases, a small surgical procedure may improve eye lubrication. Stopping an offending medication will likely result in improvement of dry eyes.

Complication: (AEU 4)

You develop dry eyes as a result of medical therapy. The dry eyes are limiting some ability to function during the day due to discomfort and visual changes. Multiple eye drops are necessary to improve the symptoms. The dry eyes will likely improve if the offending medication is stopped.

**Diarrhea:**

Diarrhea is defined as loose, watery and possibly more-frequent bowel movements. In most cases, diarrhea lasts a couple of days. But when diarrhea lasts for weeks, it can indicate a serious disorder, result in dehydration, and may require hospitalization.

Complication: (AEU 7)

You develop diarrhea as the result of medical therapy. You are experiencing 7 or more stools per day more than typical for you. At this level, you may experience incontinence and require hospitalization due to dehydration and risk for electrolyte (blood salts) abnormalities in blood. The diarrhea will likely improve over time with discontinuing the offending medication.

**Suicidal Thoughts:**

Suicide is defined as taking your own life. Suicidal thoughts can range from increased thoughts of death to intent with specific plans to carry out suicide. Risk factors for suicide include feelings of hopelessness, severe stress, psychiatric conditions such as depression and bipolar disease, and substance abuse. Treatment for suicidal thoughts may include talk therapy, psychiatric medications, and admission to the hospital. Medication adverse event suicidal thoughts may improve with stopping the offending medication but may also require some of the therapies above depending on severity.

Complication: (AEU 5)

You develop suicidal thoughts as a result of medical therapy. Thoughts may include increased thoughts of your own death. However, you do not have a specific plan or intent to actually commit suicide currently. These thoughts may improve with stopping the offending medication but may also require medical or talk therapies.

**Dizziness (Vertigo)**

Dizziness that creates the false sense that you or your surroundings are spinning or moving is called vertigo. Frequent dizzy spells or constant dizziness can significantly affect your life. But dizziness rarely signals a life-threatening condition. Dizziness may be triggered or worsened by walking, standing up or moving your head. Dizziness may accompanied by nausea or be so sudden or severe that you need to sit or lie down. Episodes can last seconds or days and may recur. Dizziness that develops as a complication of medical therapy is likely to improve with stopping the offending medication.

Complication: (AEU 5)

You develop moderate dizziness as a result of medical therapy. The symptoms are bothersome, may be associated with nausea, imbalance, and falls. The dizziness is impacting your ability to carry out your typical home and work routine. Medical therapy may be indicated. The dizziness will likely improve with stopping the offending medication.

**Pancreatitis:**

Pancreatitis is inflammation in the pancreas, an organ in the upper abdomen. Symptoms of pancreatitis may include fever, abdominal pain, nausea, and vomiting. Pancreatitis may result in an infection, kidney failure, diabetes, and collections of fluid in the abdomen. Treatment may range from stopping intake of food for a number of days, pain medications, IV fluids, and abdominal surgery. Severe cases will require hospitalization. Medication induced pancreatitis may improve with stopping offending medication and some of the treatments mentioned.

Complication: (AEU8)

You develop pancreatitis as a result of medical therapy. You develop severe abdominal pain, fever, and vomiting. Admission to the hospital is necessary. You will be treated with combination of pain medication, intravenous (IV) fluids, and nutritional support. This condition will likely improve with treatment and stopping the offending medication.

**Anemia (Low Red Blood Cells):**

Anemia is a condition in which you don't have enough healthy red blood cells to carry adequate oxygen to the body's tissues. Having anemia may make you feel tired, weak, short of breath, and dizzy. When mild, you may have no symptoms. Treatment of anemia caused by a medication may include iron supplementation, other vitamin supplementation, blood transfusion when severe, and stopping the offending medication. Most anemia caused by a medication will improve over time with stopping the offending medication.

Complication: (AEU4)

You develop anemia as a result of medical therapy. At this level of anemia, you may experience mild fatigue and shortness of breath when exercising. You may require iron or vitamin supplementation. You will NOT need a blood transfusion. This anemia is likely to improve with treatment or removal of the offending medication.

**INR Elevation**

The INR blood test is used as one measure of a person’s ability to form a normal blood clot after an injury such as a cut in your skin. An increase in the INR indicates that it is harder for blood to clot. Extreme elevations in the INR may indicate a risk to develop difficult to control bleeding. The bleeding can occur either inside the body (such as in the abdomen) or with a cut on the surface of skin. An abnormal INR can be corrected if needed with administration of medications if one experiences bleeding or has a lab value that suggests a very high risk for bleeding.

Complication: (AEU 3)

You develop a mild elevation in your INR as a result of medical therapy. At this level there is a slightly increased risk for bleeding but a serious bleeding complication is very unlikely. You may require additional bloodwork to monitor the INR level. No medications are recommended. Stopping the offending medication will likely result in returning to a normal level.

**Low White Blood Cells (Neutropenia)**

Neutropenia is a low level of a specific white blood cell, the neutrophil, which is important to prevent infections. Low neutrophil levels increase your risk to develop infections. Without infection, most patients with neutropenia will be asymptomatic. Development of a fever or infection while neutrophil levels are low may result in hospitalization and treatment with intravenous antibiotics. Severe neutropenia can be life-threatening. Medication induced neutropenia is likely to improve with removal of offending medication. Some patients may need medicines to stimulate blood cell regeneration.

Complication: (AEU8)

You develop neutropenia (low neutrophil white blood cells) as a result of medical therapy. At this level, your risk of developing an infection is moderately increased. Development of a fever may be managed with you at home. However, severe fever or development of an infection may require treatment at the hospital. The low neutrophil count may improve with removing the offending medication.

**Leg and Arm Swelling (Edema)**

Edema is swelling caused by excess fluid trapped in your body's tissues. Although edema can affect any part of your body, it's most commonly noticed in the hands, arms, feet, ankles and legs. Edema may result in swollen skin, shiny skin, skin that dimples after being pressed, and increase in abdomen size. Mild edema usually improves with raising the affected limb higher than the heart or with compression. More severe edema may require drug therapy that helps your body produce more urine.

Complication: (AEU3)

You develop moderate edema as a result of medical therapy. Swelling in your legs is noticed by you and others. No medical complications are expected because of this edema. Use of tight compression socks or a low dose of medication is likely to improve the edema significantly. Stopping the offending medication will also improve edema with time.

**Constipation:**

Constipation refers to a decrease in ability to have bowel movements, hard stools, and reduced frequency of bowel movements. Constipation may result in abdominal pain and pain when having a bowel movement. Treatment may include dietary changes and use of stool softeners, laxatives, and enemas. Medication induced constipation is likely to improve with a combination of the above treatments or stopping the offending medication.

Complication: (AEU6)

You develop severe constipation as a result of medical therapy. Due to many days without a bowel movement, you require some evacuation by a medical professional in addition to treatment with an enema, laxatives, and stool softeners. Daily activity is impacted by the constipation. The constipation is likely to improve with stopping the offending medication.

**Headache:**

Headaches may include syndromes that cause discomfort on the head including throbbing pain, stabbing pain, and numbness. Severe headaches may result in impaired physical and cognitive function. Severe headaches can impair daily function and may require treatment with medications. Drug induced headaches are likely to improve with stopping an offending medication.

Complication: (AEU 6)

You have developed a severe headache that limits routine daily activities, work, and self-care as a result of medical therapy. This headache lasts more than 1 week, may require a course of pain medication (such as ibuprofen), and may improve with discontinuing the offending medication. No ongoing medical therapy is required once headache resolves.

**Glaucoma:**

Glaucoma is a group of eye conditions that can impair vision due to increased pressure in the eye. There may be no warning signs that this condition is developing due to gradual changes in vision. This condition can affect each eye differently. Vision loss due to glaucoma is permanent. If glaucoma is discovered before it becomes severe and cause of glaucoma eliminated, remaining vision in the eye can be preserved.

Complication: (AEU 9)

You have developed severe glaucoma in at least 1 eye as a result of medical therapy. You have legal blindness in the more affected eye (visual acuity of 20/200 or worse). The vision loss in this eye is not reversible with stopping the offending medication.

**Nausea:**

Nausea is the unpleasant sensation that one needs to vomit. Nausea often results in a loss of appetite and may cause decreased intake of food and water. In severe cases, nausea can result in repeated episodes of vomiting and can place you at risk for dehydration and inadequate intake of nutrients.

Complication: (AEU 6)

You develop nausea and vomiting as a result of a medical therapy. Due to vomiting, intake of nutrients and water is reduced and you are at risk for dehydration. Treatment with anti-nausea medications may improve symptoms. The nausea and vomiting will improve with stopping the offending medication.

**Trouble Walking:**

Trouble walking can develop from a combination of limb pain, imbalance, dizziness, numbness, and weakness. Mild trouble walking will result in mild change in gait or slowed pace of walking. As trouble walking worsens, one may require the use of a cane, walker, and in severe cases wheelchair for mobility. Walking trouble as the result of a medication may improve if the offending medication is stopped.

Complication: (AEU 5)

You experience mild trouble with walking as a result of medical therapy. Walking is noticeably different due to limping, a wider based stance, or other change. Your walking speed decreases. Use of a cane, walker, or other device is not needed. Walking will likely improve with stopping the offending medication.

**Allergic Reaction**

A medication allergic reaction is an abnormal response of your immune system to a medication. The allergic reaction may cause you to develop a rash, hives, and itching. When severe, an allergic reaction can develop into anaphylaxis. Anaphylaxis is a life-threatening reaction with closing of airways, severe trouble breathing, swelling in the throat, drops in blood pressure. An anaphylaxis reaction is a medical emergency and requires treatment at a hospital. Medication allergic reactions will improve if the offending medication is stopped.

Complication: (AEU 5)

You experience an allergic reaction as the result of a medical therapy. You experience a combination of rash, hives, itching, and mild shortness of breath. Treatment with an oral and possibly intravenous (IV) medication is required. The allergic reaction improves quickly with treatment. Medications to prevent the allergic reaction from coming back are needed for less than 24 hours.

**Avascular Necrosis of a Joint:**

Avascular necrosis results in bone death due to interruption of the blood supply to the bone. This can result in breaks to the bone and collapse of the bone or joint. The condition may result in pain that ranges from mild to severe. In severe cases, if the bone collapses, surgery for a bone transplant or joint replacement may be needed.

Complication: (AEU 8.5)

You develop severe avascular necrosis of your hip as the result of a medical therapy. You experience moderate to severe pain in the hip joint. This condition results in trouble walking and inability to fully perform daily care activities. A surgical joint replacement is indicated.

**Glaucoma:**

Glaucoma is a group of eye conditions that can impair vision due to increased pressure in the eye. There may be no warning signs that this condition is developing due to gradual changes in vision. This condition can affect each eye differently. Vision loss due to glaucoma is permanent. If glaucoma is discovered before it becomes severe and cause of glaucoma eliminated, remaining vision in the eye can be preserved.

Complication: (AEU 8)

You have developed moderate to severe glaucoma in at least 1 eye as a result of medical therapy. Your doctor detects severe elevated pressure in your eye. You have moderate to severe loss of your peripheral vision. Central vision is not yet affected. Multiple eye drops, oral medications, and an eye surgery are indicated. The vision loss in this eye is not reversible with stopping the offending medication.

**Osteoporosis:**

Osteoporosis causes bones to become weak and brittle. Bone fractures, particularly in the spine or hip, are the most serious complication of osteoporosis. Treatment may include exercise, vitamin supplements, oral medications, and in severe cases injectable treatments.

Complication: (AEU 7)

You have developed osteoporosis as a result of medical therapy. Weakness in multiple bones is discovered with x-ray testing. You experience a bone fracture that does not require surgery or an admission to the hospital. The fracture may result in pain and require a splint or cast for some period of time. At this level, you may be at risk for other fractures in the future. Removing the offending medication may slow the progression of osteoporosis but will not allow you to regain bone density.

**Stroke:**

A stroke occurs when the blood supply to part of your brain is interrupted or severely reduced, depriving brain tissue of oxygen and nutrients. Within minutes, brain cells begin to die. As a result of the stroke, patients can develop paralysis of arms or legs, trouble talking and swallowing, sensory loss in the limbs, thinking dysfunction. When deficits are severe, some patients must be fed through a tube in the stomach and may need to have 24 hour care at home or in a nursing home following a stay in the hospital.

Complication: (AEU 6)

You develop a mild stroke as a result of medical therapy. You may have mild symptoms such as mild hand weakness or numbness in your arm and leg due to this stroke. It is also possible that this stroke causes no symptoms and is detectable only with an image of your brain. The stroke will result in no long –term deficits. However, this stroke increases your risk of a future, larger stroke. You may require new medications to reduce your risk of a future stroke.

**Stomach Ulcer:**

Stomach ulcers are sores that can develop on the inner surface of your stomach or intestines. These symptoms can result in stomach pain, heartburn, and intolerance of some foods. When severe, the ulcers can result in loss of blood resulting in dark colored stools, dizziness, fatigue, and weight loss. Treatment may include medications to reduce stomach acid production, antibiotics, and in rare cases, surgery. Ulcers caused by medications may improve over time if the offending medication is stopped.

Complication: (AEU 4)

You develop a mild stomach ulcer as the result of medical therapy. You have no symptoms because of the ulcer. Routine follow-up, lab tests, and possibly imaging of the stomach is likely recommended. No medications or other treatments are recommended. If treatment with the offending medication continues, it is possible that the ulcer could worsen with time. It is likely the ulcer will heal if the offending medication is stopped.

**Pulmonary Fibrosis**

Pulmonary fibrosis is a lung disease that occurs when lung tissue becomes damaged and scarred. This thickened, stiff tissue makes it more difficult for your lungs to work properly. As pulmonary fibrosis worsens, you become progressively more short of breath. The lung damage caused by pulmonary fibrosis can't be repaired. Complications from this condition can shorten life expectancy. Medications and therapies can sometimes help ease symptoms and improve quality of life. For some people, a lung transplant might be appropriate.

Complication: (AEU 6)

You develop mild pulmonary fibrosis as a result of medical therapy. Tissue damage is discovered in less than 25% of your lung tissue based on an image of your chest. You may develop mild shortness of breath with activity due to low oxygen levels. This level of pulmonary fibrosis will not improve but may stop progressing with stopping the offending medication.

**Weight Gain:**

Weight gain can result in moving from a status of normal weight to overweight or obese. Obesity isn't just a cosmetic concern. Developing obesity increases your risk of diseases and health problems, such as heart disease, diabetes and high blood pressure. Discontinuation of medicine may not be enough to lose gained weight.

Complication: (AEU 3)

You have developed a mild increase in your weight (5-10% increase from baseline) as a result of medical treatment. This amount of weight gain may affect your appearance but is unlikely to result in increasing your risk of disease and health problems. This degree of weight gain will likely improve with stopping the medication and changes in diet and activity.

**Diarrhea:**

Diarrhea is defined as loose, watery and possibly more-frequent bowel movements. In most cases, diarrhea lasts a couple of days. But when diarrhea lasts for weeks, it can indicate a serious disorder, result in dehydration, and may require hospitalization.

Complication: (AEU 3)

You develop mild diarrhea as the result of medical therapy. You are experiencing less than 4 more stools per day more than typical for you. At this level, you are not likely to develop dehydration or other medical complications of diarrhea. The diarrhea will likely improve over time with discontinuing the offending medication.

**Hallucinations**Hallucinations are the symptom of seeing or hearing things that don't exist. For the person experiencing this symptom, the hallucinations have the full force and impact of a normal experience. Hallucinations can be in any of the senses, but hearing voices is the most common hallucination. This symptom can impact the ability to function in day to day life. In severe cases, patients who develop this symptom may require treatment with anti-psychotic medications and psychotherapy. Medication induced hallucinations are likely to improve with stopping the offending medication.

Complication: (AEU 5)

You have developed mild hallucinations as a result of medical therapy. The symptoms are noticeable but do not affect daily life activities or require treatment with anti-psychotic medications. Symptoms are likely to improve if offending medication is stopped.

**Abnormal Movements (Dyskinesia):**

Abnormal, involuntary movements of limbs or head can develop as a result of medical therapies. These movements can include movements of the tongue, lip puckering, movement of arms, foot tapping, shoulder shrugging. The movements vary from unnoticeable to others to severe, violent movements. The movements may or may not be reversible when offending medication is stopped.

Complication: (AEU 6)

You develop severe restlessness or severe increase in uncontrollable limb movements as a result of medical therapy. These symptoms limit your ability to take care of yourself and significantly impair your daily activities. The symptoms may or may not improve with discontinuing the offending medication.

**Heart Rhythm Disorder (Arrhythmia):**

Arrhythmias are changes to the electrical system that controls your heart rate. When abnormal, your heart may beat too fast or slow. Patients with arrhythmias may experience a racing heart, chest fluttering, shortness of breath, chest pain, and lightheadedness depending on the type of abnormal rhythm and severity. Some arrhythmias place you at risk for complications such as stroke, heart attack, and heart failure. Treatment may involve medications, placement of a pacemaker or defibrillator, or minor surgical procedures. Some arrhythmias caused by a medication will improve if the offending medication is stopped.

Complication: (AEU 4)

You develop a mild heart rhythm abnormality as a result of medical therapy. The abnormal rhythm is discovered by a routine EKG (electrical test) performed by your doctor. You have no symptoms because of the heart rhythm. No medications or other treatments are recommended. This rhythm may improve with stopping the offending medication.

**Hypertension (High Blood Pressure)**

High blood pressure is a common condition in which the pressure against your artery walls is high enough that it may eventually cause health problems, such as heart disease. Even without symptoms, damage to blood vessels and your heart continues and can be detected. Uncontrolled high blood pressure increases your risk of serious health problems, including heart attack and stroke. High blood pressure caused by a medication side effect may or may not improve with removing the offending medication.

Complication: (AEU 5)

You develop moderate high blood pressure as a result of medical therapy. Your high blood pressure is consistently higher than 160/100. Prolonged exposure to this level of high blood pressure will increase your risk of heart attack, stroke, heart failure, and other complications of high blood pressure. You will require treatment with a medication to lower blood pressure. Treatment with high blood pressure medications and removal of the offending medication will likely improve your blood pressure.

**Kidney Failure**

Kidney failure, describes the loss of kidney function. Your kidneys filter wastes and excess fluids from your blood. When chronic kidney disease reaches an advanced stage, dangerous levels of fluid, electrolytes and wastes can build up in your body. Complications of kidney failure may include limb swelling, heart disease, cognitive changes, fatigue, low red blood cells, increased infections, difficult to control high blood pressure. In severe cases, patients may require a kidney transplant. Kidney failure caused by medication side effect may improve with stopping the offending medication.

Complication: (AEU 5)

You develop mild kidney failure as a result of medical therapy. The kidney failure is detected with a routine blood test only. You have no symptoms. You may require periodic blood tests to monitor your kidney function to be sure it isn’t getting worse. At this level, stopping the offending medication is likely to result in improvement in kidney failure.

**Myalgia (Muscle Pain):**

Myalgias are deep muscle aches. When severe, pain may impair daily function. Treatment with pain medications may relieve the symptom. Myalgia caused as an adverse effect of medical treatment will likely improve when the offending medication is stopped.

Complication: AEU 3

You develop mild myalgia (muscle pain) as a result of medical therapy. The pain is noticeable but does not limit routine daily activities, self-care, or exercise ability. You may or may not require mild pain medications, like Tylenol(acetaminophen to help relieve the symptoms. The pain is likely to improve with stopping the offending medication.

**Seizure:**

A generalized seizure features a loss of consciousness and violent muscle contractions. On the other hand, focal seizures can result in abnormal movement of a limb with normal consciousness. Seizures are caused by abnormal electrical activity throughout the brain. Short lived seizures do not result in injury to the brain. Prolonged seizures that last for 30 or more minutes may result in some changes to the brain. Patients who develop repeated seizures often require medical treatment to prevent additional events. Patients who experience a drug induced seizure are unlikely to experience another one if the medication is stopped.

Complication: (AEU 5)

You experience a short, focal seizure with normal consciousness as a result of medical treatment. This short seizure causes some abnormal movements of an arm or leg. This short seizure does not result in injury to the brain and does not require admission to a hospital. It is unlikely to occur again if the offending medication is stopped. Your doctor is unlikely to recommend treatment with an anti-seizure medication.

**Depression:**

Depression is a mood disorder that causes a persistent feeling of sadness and loss of interest. Depression can cause trouble doing normal day-to-day activities. When severe depression can cause one to feel that life isn’t worth living. Many people with depression feel better with medication, psychotherapy, or both.

Complication:

You have developed depression as a result of medical treatment. Discontinuing the medication may not be enough to reverse this condition. Due to depression you may be having trouble with activities such as cleaning the house, managing money, preparing meals, and your job. Treatment with anti-depressant medications and discontinuing the offending medication is likely to improve the depression.

**Fever:**

A fever is a temporary increase in your body temperature, often because of an illness. Fever seems to play a role in helping your body fight off infection. Fever can be accompanied by sweating, body aches, chills, dehydration, and loss of appetite. Fever is generally not dangerous until it reaches 103F (39.4C). Fever of this level generally requires medical attention. With improvement in the underlying cause of fever, the fever typically resolves.

Complication:

You have developed a fever of 104F(40C) for less than 1 day as a result of medical treatment. You have additional symptoms of chills, body aches, and loss of appetite. You needed to visit your doctor to discuss the fever. The fever gets better in less than 24 hours (1 day) with discontinuing the offending medication. You may have taken a medication like Tylenol or Advil to treat the symptoms of fever.

**Sexual Dysfunction:**

Persistent recurrent problems with sexual response, desire, orgasm, or pain that distress you or strain relationship with your partner are known medically as sexual dysfunction. If induced by a medication, this symptom typically improves with discontinuing the medication.

Complication:

You have developed a decrease in sexual desire and sexual response due to a medical treatment. This symptom is adversely affecting your relationship with a partner. Discontinuing the medication is likely to result in improved sexual function.

**Anxiety:**

Generalized anxiety is an ongoing feeling of worry that is difficult to control. This worry and anxiety interferes with day to day activities. Symptoms can include persistent worry out of proportion to impact of events, difficulty handling uncertainty, inability to relax, feeling restless, and difficulty concentrating. Anxiety can also result in fatigue, muscle aches, trembling, or trembling. Many people with anxiety feel better with medication, psychotherapy, or both.

Complication:

You have developed anxiety as a result of medical treatment. Discontinuing the medication may not be enough to reverse this condition. Due to anxiety you may be having trouble with activities such as cleaning the house, managing money, preparing meals, and your job. Treatment with psychotherapy, possible treatment with anti-depressant medications, and discontinuing the offending medication is likely to improve the anxiety.

**Mania:**

Mania is an feeling of an abnormal emotional high. When experiencing mania, a person can develop feelings of euphoria, excess energy, and irritability. These feelings can make it difficult to sleep for days, impair judgment, decrease ability to think clearly, and result in poor decision making. Discontinuing an offending medication may improve these symptoms. Some people will require treatment with an anti-depressant or other psychoactive medication.

Complication:

You have developed severe mania as result of medical treatment. You have been unable to sleep for days, are unable to work, are having trouble with daily activities such as cooking, have poor hygiene, and have made poor financial decisions. The symptoms require a short hospitalization. Discontinuing the offending medication and a short course of psychoactive drugs is likely to result in improvement.

**Insomnia:**

Insomnia is a common sleep disorder that can make it hard to fall asleep, hard to stay asleep, or cause you to wake up too early and not be able to get back to sleep. You may still feel tired when you wake up. When severe, lack of sleep can decrease daytime energy, affect mood, decrease work performance, and decrease quality of life.

Complication:

You develop mild difficulty falling asleep, staying asleep, or waking up early as a result of medical treatment. The symptom has been present for a couple of weeks. You feel more tired during the day than usual but are able to function normally. This symptom is likely to improve if the offending medication is discontinued.

**Fatigue:**

Abnormal fatigue results in unrelenting exhaustion that isn’t relieved by rest or causes one to seek more rest than usual. This feeling can reduce energy, motivation, and concentration. When severe it can also impact emotional and psychological well-being.

Complication:

You develop fatigue due to a medication that causes you to seek additional rest. Despite seeking additional rest, the fatigue does not completely improve. You are able to complete your daily activities. The fatigue is likely to improve with discontinuing the offending medication.

**Vascular Access Complications:**

Occasionally patients require ongoing treatment with medications that need to be infused through a vein. This can require placement of a long-term intravenous catheter or tube into a vein. These catheters can sometimes result in complications such as infections or blood clots. Severe infection can result in hospitalization and blood clots can cause other complications such as strokes or clots in the lungs. A complication due to a catheter may require removing the device and treatment with medications.

Complication:

You develop a severe infection as the result of a central vein catheter. The infection requires admission to the hospital, treatment with antibiotics, and removal of the central venous catheter. This is a life-threatening event that you are likely to survive if the infection is treated quickly. Due to the infection it is likely that a new catheter cannot be placed for a number of weeks. Your medication for which you had the catheter can be infused during this time by placing temporary IV catheters into a vein at the time of each infusion.

**Treatment Related Complication to a Child:**

Treatments administered during pregnancy can result in injury to the developing child. Treatment related complications can result in structural problems (for example incomplete fusion of the spinal bones, abnormal heart structure, brain abnormalities). Treatment related complications can also result in intellectual disabilities that range from mild to severe, physical handicaps, and in severe cases death of the child.

Complication:

As a result of a medical treatment during pregnancy, you have a child born with abnormalities of brain development. The child will have severe intellectual disabilities including trouble with understanding written language, numbers, money, and time. They will require lifelong caretakers to provide extensive support. Over time the child will have some ability to perform daily activities such as bathing, dressing, and eating. They will require help with all more complex activities.

Complication 2:

As a result of a medical treatment during pregnancy, you have a child born with a mild physical change (for example a single crease/line on the palm of the hand or a small skin tag near the ear) that does not result in any physical disability. The child will develop normally and will be able to function normally in society.

**Reproductive Dysfunction:**

Reproductive dysfunction results in difficulty getting pregnant despite having frequent unprotected sex for at least a year. This can result from reproductive dysfunction in a male or female. Rarely this symptom is accompanied by changes to the menstrual period in a female and signs of hormonal dysfunction in a male (for example changes in hair growth).

Complication:

Despite a desire to conceive a child, as a result of a medical treatment, you or your partner have been unable to get pregnant for over a year. Discontinuing the offending medication is likely to result in ability to have a pregnancy in the future.

**Urine Retention:**

Urine retention is the accumulation of urine in the bladder due to an inability to fully empty the bladder when urinating. This can result in some abdominal discomfort, recurrent feelings of an urge to urinate, and in severe cases urine infections or kidney abnormalities.

Complication:

As a result of medical treatment, you develop difficulty emptying your bladder completely when attempting to urinate. This results in some discomfort but has not resulted in an infection or kidney trouble. Discontinuing the offending medication is likely to improve this symptom.

**Appendix 4: Estimates, Standard errors and calibration plot from the logistic regression model fit to the mTurk data using Firth’s method.**

|  | Estimate from logistic regression | SE |
| --- | --- | --- |
| Diabetes | 0.82 | 0.39 |
| Osteoporosis | -0.59 | 0.43 |
| Weight Gain 6 | -0.87 | 0.38 |
| Cognitive Impairment | -0.78 | 0.38 |
| Seizure | 0.33 | 0.36 |
| Heart Attack | 1.60 | 0.39 |
| Deep Vein Thrombosis (DVT) | 0.71 | 0.41 |
| Headache | -1.57 | 0.37 |
| Hallucinations 6 | -0.29 | 0.38 |
| Treatment Related Malignancy (Cancer) | 1.94 | 0.40 |
| Myalgia (Muscle Pain) 6 | -2.04 | 0.94 |
| Itchy Skin (Pruritus) | -1.31 | 0.37 |
| Low Platelets | -1.05 | 0.38 |
| Low Blood Sodium (Hyponatremia) | -0.08 | 0.35 |
| Flu-Like Symptoms | -1.90 | 0.35 |
| Abnormal Movements (Dyskinesia) 4 | -0.43 | 0.37 |
| Kidney Stones | 0.35 | 0.36 |
| Diarrhea | -1.73 | 0.38 |
| Pulmonary Fibrosis 9 | 1.61 | 0.36 |
| Hypertension (High Blood Pressure) | -0.66 | 0.38 |
| Liver Disease | 0.39 | 0.36 |
| Kidney Failure 8 | 0.84 | 0.37 |
| Infection | 0.17 | 0.36 |
| Weight Loss | -0.45 | 0.35 |
| Dizziness (Vertigo) | -1.47 | 0.37 |
| Hair Loss | -1.05 | 0.38 |
| Headache 2 | -2.08 | 0.38 |
| Congestive Heart Failure | 1.09 | 0.41 |
| Stroke | 2.74 | 0.40 |
| Cataracts | -0.99 | 0.37 |
| Dry Eyes | -1.55 | 0.47 |
| Diarrhea 7 | -0.84 | 0.42 |
| Suicidal Thoughts | 0.51 | 0.40 |
| Dizziness (Vertigo) | -0.31 | 0.45 |
| Pancreatitis | 0.84 | 0.38 |
| Anemia (Low Red Blood Cells) | -0.73 | 0.42 |
| INR Elevation | -1.08 | 0.36 |
| Low White Blood Cells (Neutropenia) | 0.02 | 0.36 |
| Leg and Arm Swelling (Edema) | -1.13 | 0.36 |
| Constipation | -0.94 | 0.37 |
| Headache 6 | -1.06 | 0.37 |
| Glaucoma 9 | 0.53 | 0.35 |
| Nausea | -1.18 | 0.38 |
| Trouble Walking | -0.73 | 0.41 |
| Allergic Reaction | -1.10 | 0.40 |
| Avascular Necrosis of a Joint | 1.12 | 0.36 |
| Glaucoma 8 | 0.67 | 0.37 |
| Osteoporosis | 0.29 | 0.36 |
| Stroke 6 | 0.63 | 0.44 |
| Stomach Ulcer | -0.43 | 0.37 |
| Pulmonary Fibrosis 6 | 0.83 | 0.37 |
| Weight Gain 3 | -1.51 | 0.36 |
| Diarrhea 3 | -2.39 | 0.43 |
| Hallucinations 5 | -0.59 | 0.36 |
| Abnormal Movements (Dyskinesia) 6 | 0.34 | 0.37 |
| Heart Rhythm Disorder (Arrhythmia) | -0.34 | 0.37 |
| Hypertension (High Blood Pressure) | -0.03 | 0.38 |
| Kidney Failure 5 | 0.10 | 0.37 |
| Myalgia (Muscle Pain) 3 | -1.70 | 0.36 |
| Depression | -0.49 | 0.36 |
| Fever | -1.88 | 0.37 |
| Sexual Dysfunction | -1.75 | 0.38 |
| Anxiety | -0.79 | 0.37 |
| Mania | 0.21 | 0.39 |
| Insomnia | -1.50 | 0.37 |
| Fatigue | -1.96 | 0.40 |
| Vascular Access Complications | 1.32 | 0.39 |
| Teratogen 1 | 2.30 | 0.39 |
| Teratogen 2 | 0.00 | 0.29 |
| Reproductive Dysfunction | -1.24 | 0.37 |
| Urine Retention | -1.37 | 0.38 |
| Heart Failure | 1.82 | 0.61 |
| Neutropenia | Reference (0) | -- |

Calibration plot from the logistic regression model fit to the mTurk data using Firth’s method


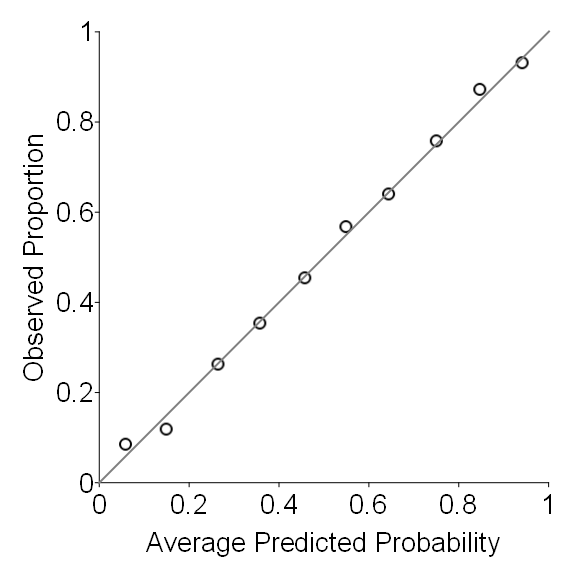


**Appendix 5: Results of K-means clustering applied to mTurk preference parameters**


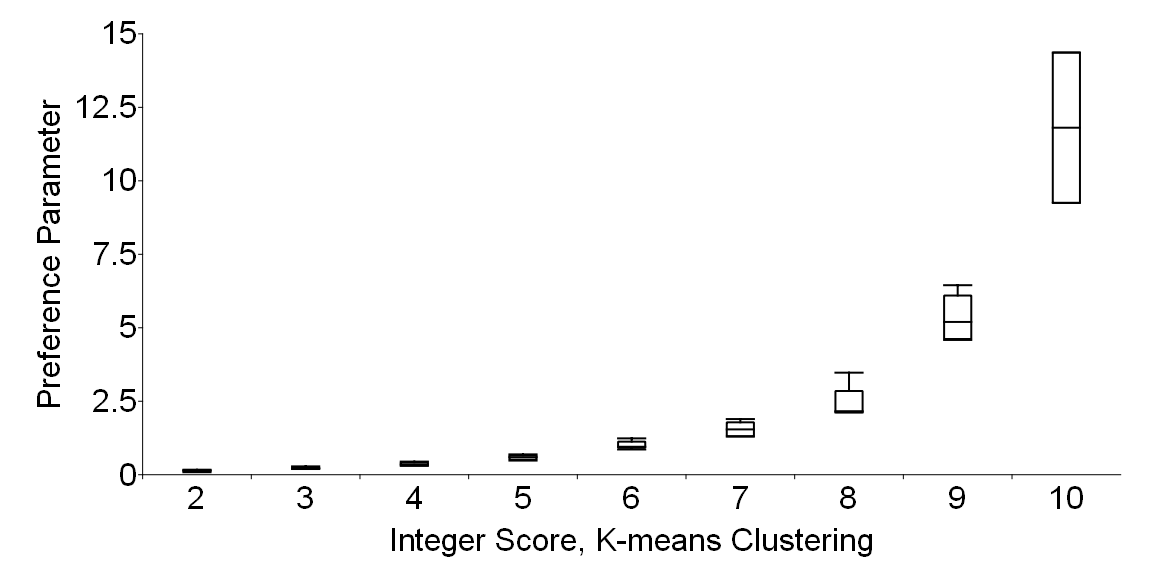

Supplement: S1 File — (DOCX) [file pone.0262109.s001.docx]
